# Absence of claudin-3 does not alter intestinal absorption of phosphate in mice

**DOI:** 10.1007/s00424-024-02998-x

**Published:** 2024-08-08

**Authors:** Zsuzsa Radványi, Udo Schnitzbauer, Eva Maria Pastor-Arroyo, Simone Hölker, Nina Himmerkus, Markus Bleich, Dominik Müller, Tilman Breiderhoff, Nati Hernando, Carsten A. Wagner

**Affiliations:** 1 Institute of Physiology, University of Zurich, Zurich, Switzerland; 2 Institute of Physiology, Kiel University, Kiel, Germany; 3 Department of Pediatrics, Division of Gastroenterology, Nephrology and Metabolic Diseases, Charité-Universitätsmedizin Berlin, Berlin, Germany

**Keywords:** Intestinal absorption, Phosphate, Calcium, Cldn3, Paracellular transport, Vitamin D3

## Abstract

Intestinal absorption of phosphate is bimodal, consisting of a transcellular pathway and a poorly characterized paracellular mode, even though the latter one contributes to the bulk of absorption under normal dietary conditions. Claudin-3 (*Cldn3*), a tight junction protein present along the whole intestine in mice, has been proposed to tighten the paracellular pathway for phosphate. The aim of this work was to characterize the phosphate-related phenotype of *Cldn3*-deficient mice. *Cldn3*-deficient mice and wildtype littermates were fed standard diet or challenged for 3 days with high dietary phosphate. Feces, urine, blood, intestinal segments and kidneys were collected. Measurements included fecal, urinary, and plasma concentrations of phosphate and calcium, plasma levels of phosphate-regulating hormones, evaluation of trans- and paracellular phosphate transport across jejunum and ileum, and analysis of intestinal phosphate and calcium permeabilities. Fecal and urinary excretion of phosphate as well as its plasma concentration was similar in both genotypes, under standard and high-phosphate diet. However, *Cldn3*-deficient mice challenged with high dietary phosphate had a reduced urinary calcium excretion and increased plasma levels of calcitriol. Intact FGF23 concentration was also similar in both groups, regardless of the dietary conditions. We found no differences either in intestinal phosphate transport (trans- or paracellular) and phosphate and calcium permeabilities between genotypes. The intestinal expression of claudin-7 remained unaltered in *Cldn3*-deficient mice. Our data do not provide evidence for a decisive role of *Cldn3* for intestinal phosphate absorption and phosphate homeostasis. In addition, our data suggest a novel role of *Cldn3* in regulating calcitriol levels.

**Supplementary Information:**

The online version contains supplementary material available at 10.1007/s00424-024-02998-x.

## Introduction

Absorption of inorganic phosphate (Pi) in the gut takes place mostly along the small intestine. The molecular type and content of dietary phosphate depend on the dietary source. Processed food contains high levels of bioavailable inorganic phosphate (Pi), whereas phosphate comes mostly in the form of organic esters or phytate in unprocessed food from animal or plant origin, respectively (for review, see [28]). Regardless of the source, dietary phosphate must be hydrolyzed for Pi to be absorbed. Thus, organic esters are hydrolyzed in the gastrointestinal tract by the action of the alkaline phosphatase, whereas hydrolysis of phytate would require phytase, an enzyme that is not expressed in mammals [33]. Therefore, the source of phosphate critically controls its bioavailability. Upon intestinal absorption and release into the blood stream, Pi in excess is excreted into urine. Therefore, in states of compromised renal function, such as chronic kidney disease (CKD), high phosphate consumption may exceed the renal capacity to compensate for intestinal absorption, thus resulting in hyperphosphatemia. High plasma Pi is known to associate with cardiovascular disease and mortality not only in patients with CKD [1, 3, 19, 34] but also in the normal population [6, 8, 28]. Since the content of phosphate in Western food is well above the levels recommended by the World Health Organization, it is critical to understand the mechanisms mediating intestinal absorption of Pi, as this step has classically received less attention than the renal counterpart.

Intestinal absorption of Pi proceeds via two independent pathways, namely, a passive route the capacity of which is proportional to the luminal concentration of Pi and an active component that gets saturated with increasing luminal concentrations of the substrate [18, 40]. This last component involves uptake of luminal/dietary Pi across the brush border membrane (BBM) of enterocytes, a step mediated by NaPi-IIb/*Slc34a2* [13, 21], followed by efflux of Pi across the basolateral membrane via a transporter whose identity remains to be unambiguously defined (for review, see [15]). By analogy with other ions, the passive component is expected to proceed paracellularly across tight junctions [36]. In all intestinal segments of rats and mice, the luminal concentration of Pi is well above the plasma levels (5.7–35 mM vs. 1–2 mM), thus providing the driving force for passive transport [20, 25].

NaPi-IIb localizes to the BBM of enterocytes in the small intestine [16], and though it mediates most if not all active transport of Pi in the gut, its absence in mice has very modest consequences in overall intestinal absorption of the anion [13, 30]. This observation, together with the fact that the K_m_ for Pi of NaPi-IIb (≈50 µM) is far below the intraluminal Pi concentration [25], suggests that the passive route mediates the bulk of Pi absorption. In mice, all intestinal segments show a passive Pi conductance, since transport activity increases with increasing intraluminal concentrations of Pi and the direction of transport follows the gradient of the ion [36]. Unlike the active component, passive Pi transport is not regulated by vitamin D_3_ [14]. Still, the identity of the tight junction protein(s) responsible for the formation of the Pi paracellular pathway remains unknown. It has been reported that lithocholic acid, a vitamin D receptor (VDR) agonist, increases Pi transport across the whole intestine of mice in a VDR-dependent but Na^+^-independent manner [11]. Lithocholic acid reduced the intestinal expression of claudin-3/*Cldn3* and occludin, two tight junction components, in a VDR-dependent manner, but without changing the expression of NaPi-IIb. Moreover, everted sacs from *Cldn3* knockouts (KO) showed an increased transport for Pi when exposed to intraluminal Pi concentrations of 1.2 mM. Also, the Pi concentration in hepatic bile is increased in this mouse model [37]. Based on these observations, it has been suggested that claudin-3 may tighten the paracellular pathway for Pi.

Here, we have analyzed the effect of *Cldn3* deletion on intestinal absorption and transport routes of Pi and on several parameters related to Pi metabolism in mice under standard conditions as well as upon a challenge with high Pi diets. Our results do not support a role of claudin-3 in intestinal absorption of Pi.

## Material and methods

### Ethical approval

All animal experiments were approved by the local veterinary authority, Kantonales Veterinäramt Zürich (ZH240/2019), and performed according to Swiss Animal Welfare laws.

### Animal and tissue handling

Generation of global constitutive *Cldn3* knockout (KO) mice has been previously described [23]. Mice heterozygous for the deleted allele (*Cldn3*^+/−^) were bred to obtain homozygous *Cldn3*^−*/*−^ (KO) and *Cldn3*^+*/*+^ (wild type, WT) littermates that were identified by genotyping as reported. Mice were kept at a standard 12 h light and dark cycle with free access to food and water. Experiments were performed with 3-month-old males.

In a first experiment, *Cldn3* KO and WT littermates fed ad libitum on standard chow (0.6% Pi, 1% Ca, 1000 IU Vit. D3; ssniff, Soest, Germany) were adapted individually for 24 h to metabolic cages (Tecniplast, Italy), upon which 24 h urine and feces were collected. In a second experiment, mice were also placed in metabolic cages but received ad libitum high Pi diet (1.2% Pi, 1% Ca, 1000 IU Vit. D3; ssniff, Soest, Germany) for a total duration of 3 days, with collection of the last 24 h urine. In both experiments, at the end of the 24 h collection, mice were anaesthetized with vaporized isoflurane for terminal organ extraction, and venous blood was collected with heparinized syringes. Plasma was separated from total blood by centrifugation, aliquoted, and stored at − 80 °C. The animals were then sacrificed by cervical dislocation and their kidneys and intestine harvested. Freshly isolated intestinal segments were flushed with 0.9% NaCl solution, and the middle part of jejunum and ileum was transferred immediately to standard Ringer solution (in mM: 145 NaCl, 3.6 KCl, 3.6 NaHCO_3_, 5 glucose, 1 MgCl_2_·6 H_2_O, 1.3 Ca-gluconate·H_2_O, pH 6.0). These segments were then cut open longitudinally, inserted into tissue holders, and mounted into Ussing chambers for measurement of radioactively labeled (^32^P) Pi flux and of dilution potentials as described previously [14, 20]. The rest of the intestinal tissue (duodenum, remains of jejunum, and ileum, as well as proximal and distal colon) was inverted and rinsed with 0.9% NaCl prior collection of the luminal mucosa by scraping; scrapings were stored at − 80 °C. Urine samples were centrifuged at room temperature to remove debris and stored at − 20 °C. Stool samples were dried at 80 °C over the course of 3 days and dissolved in 0.6 M HCl (100 mg/ml) as reported [16]. Upon homogenization and centrifugation, supernatants were kept at room temperature.

### Quantification of ions in plasma, stool, and urine

Fecal and urinary Pi content was colorimetrically determined (Randox kit, UK) according to the Fiske-Subbarow method [7], whereas the amount of Ca^2+^ in stool and urine samples was measured with the QuantiChrom Calcium Assay Kit (BioAssay Systems). The rest of the urinary (Na^+^, K^+^, Mg^2+^, and Cl^−^) and all plasma ions were measured in a SYNCHRON® System (Beckman Coulter) at the Zürich Integrative Rodent Physiology facility. Fecal excretions were normalized to 24 h food intake, whereas urinary parameters were corrected by the urinary creatinine concentration measured by the Jaffe method.

### Quantification of plasma FGF23, PTH, and calcitriol

The amount of intact fibroblast growth factor 23 (FGF23) as well as of intact parathyroid hormone (PTH) in plasma was determined by ELISA (Immutopics International) according to the manufacturers’ protocol. The levels of calcitriol were measured with a radioimmunoassay kit (Immunodiagnostic System).

### Flux measurements

Pi (^32^P) fluxes across the jejunum and ileum were examined in Ussing chambers as previously described [20]. Briefly, freshly isolated intestinal segments were mounted on tissue holders and inserted into Ussing chambers and then equilibrated for 30 min with standard Ringer solution (continuously oxygenated and kept at 37 °C with a water jacket) on both sides of the tissue. Upon equilibration, either a 100 µM or a 70 mM apical-to-basolateral Pi gradient was established by the addition of NaH_2_PO_4_/^32^P solution to the chamber in contact with the luminal side of the tissue (“apical chamber”), whereas the Ringer added to basolateral side contained no Pi. The 100 µM Pi gradient is a condition where active transport is preferred, whereas a concentration gradient of 70 mM Pi favors the passive transport of the anion. Aliquots were taken from the basolateral side 0, 60, 90, and 120 min after the addition of radiolabeled ^32^P to the apical side. At each time point, apical aliquots of the same volume were also discarded to avoid changes in hydrostatic pressure. In a second set of experiments, jejunal segments were subjected to either a 2 mM or a 70 mM Pi gradient (NaH_2_PO_4_/^32^P) both from the apical to basolateral (AP to BL) and basolateral to apical (BL to AP) sites, and fluxes measured are described above. The amount of ^32^P transported from the donor to the acceptor side was quantified with a β-counter (Packard BioScience).

### Dilution potential measurements

Dilution potential measurements were performed in freshly isolated segments of the jejunum and ileum from mice fed 3 days on high Pi diet as reported [20]. Tissues were mounted in modified Ussing chambers (exposed area of 0.076 cm^2^) with a flow-through system, where the apical and basolateral sides were independently and continuously perfused with a flow rate of 5–10 ml/min. After equilibration, one side of the chamber was consecutively perfused with either a standard Ringer solution (145 mM NaCl, pH 6), a low NaCl Ringer (30 mM NaCl supplemented with 230 mM mannitol, pH 6), a high Pi Ringer (140 mM NaH_2_PO_4_, pH 6), or a high CaCl_2_ Ringer (72.5 mM CaCl_2_ supplemented with 75 mM mannitol, pH 6). Solutions were applied first to the apical and then basolateral chamber, while the other chamber was simultaneously perfused with standard Ringer solution. Differences in transepithelial voltage (*V*_te_) upon changes of perfusion solution were measured under open-circuit conditions and corrected for previously defined liquid junction potentials, with *V*_te_ referring to the apical side. Transepithelial resistance (*R*_te_) was assessed with short pulses (1 s) of 1.22 µA, and the difference in *V*_te_ upon the pulse was used to calculate the *R*_te_ according to Ohm’s law (*R*_te_ = Δ*V*_te_/*I*) after correction for the resistance of the empty chamber. Relative and absolute permeabilities for monovalent ions (Na^+^, Cl^−^, and Pi) were calculated using the Goldman-Hodgkin-Katz equation and *R*_te_, while permeability values for divalent Ca^2+^ were calculated with the simplified Kimizuka-Koketsu equation. Permeability ratios (*P*_Na_/*P*_Cl_, *P*_Na_/*P*_Pi_, and *P*_Na_/*P*_Ca_) were obtained by the Goldman-Hodgkin-Katz equation, and *R*_te_ was used to calculate absolute permeabilities.

### Isolation of total membrane fractions from the ileum and jejunum

Ileal and jejunal mucosal scrapings were placed in homogenization buffer consisting of 200 mM mannitol, 41 mM KOH, and 80 mM HEPES (pH 7.5), supplemented with protease/phosphatase inhibitors (Roche, Switzerland). Upon addition of MagNa Lyser Green Beads (Roche, Switzerland), tubes were placed in a Precellys 24 homogenizer (Bertin Instruments) and subjected to two rounds of 6800 rpm, 30 s each. Samples were then centrifuged at 6000 rpm for 20 min at 4 °C. Supernatants were further centrifuged (41,000 rpm, 30 min at 4 °C), and the pellets containing total membrane proteins were resuspended in the homogenization buffer by sonication.

### Renal brush border membrane preparation

Renal brush border membranes (BBM) were isolated from frozen kidneys as described in detail [2]. Half of a kidney was homogenized with a Polytron PT 10–35 (Kinematica GmbH) in a buffer containing 300 mM mannitol, 5 mM EGTA, and 12 mM Tris–HCl (pH 7.1), supplemented with protease/phosphatase inhibitors (Roche, Switzerland). Upon addition of MgCl_2_ (final concentration 12 mM) and 15 min precipitation on ice, the samples were centrifuged (4500 rpm, 15 min, 4 °C), and the supernatants further centrifuged at 17,500 rpm, 30 min at 4 °C. Pellets containing the BBMs were resuspended in a buffer containing 300 mM mannitol, 20 mM HEPES-Tris, pH 7.4.

### Protein determination and Western blot

The protein concentration of ileal and jejunal total membrane fractions and renal BBMs was measured with the Bio-Rad DC protein assay kit. Aliquots containing 20 µg protein were mixed with Laemmli sample buffer, separated on 9% or 12% SDS/PAGE gels, and transferred onto polyvinylidene difluoride (PVDF) membranes (Millipore, Switzerland). Membranes were then blocked with 5% fat-free milk powder in Tris-buffered saline (TBS) for 30 min prior to overnight incubation at 4 °C with primary antibodies against NaPi-IIa (1:3000) [5], NaPi-IIb (1:2000) [25], claudin-2 (1:300, #51–6100 Thermo Fisher), claudin-3 (1:1000, #34–1700 Invitrogen, Switzerland), claudin-7 (1:1000, #34–9100 Invitrogen), calbindin-D28k (1:500, #CB38 Swant), VDR (1:300, #sc-13133 Santa Cruz Biotechnology), or β-actin (1:15,000, #A1978 Sigma-Aldrich, Switzerland). Upon washing with TBS, membranes were incubated for 1 h at room temperature with the appropriate HRP-conjugated secondary antibodies (Promega, Switzerland). After further washing with TBS, membranes were exposed to HRP substrate (Western Chemiluminescence HRP Substrate, Millipore, Switzerland) for 5 min before the chemiluminescent signal was detected with a LAS-4000 camera system (Fujifilm). The densitometric analysis was performed with the Image Studio Lite program, and the density of the proteins of interest was normalized to β-actin.

### Immunofluorescence of intestinal segments

Intestinal rings of freshly dissected duodenum, jejunum, ileum, and proximal colon were fixed in 4% PFA in PBS, incubated in increasingly concentrated solutions of sucrose in PBS, then embedded in optimal cutting temperature compound (OCT, Tissue-Tek, Sakura, USA), and frozen in liquid propane. For immunostainings, 1–5 µm thick cryosections were cut and mounted on Superfrost Plus Slides (Thermo Scientific, Switzerland) and then rehydrated in PBS for 30 min. Samples were incubated 5 min in 1% SDS in PBS for antigen retrieval, upon which the sections were washed in PBS and blocked with 1% BSA in PBS for 15–30 min. Incubation with the primary antibody of Claudin-3 (1:50, #34–1700 Invitrogen, Switzerland) or of Claudin-7 (1:200, #34–9100 Invitrogen, Switzerland) was done overnight at 4 °C. Upon washes with hypertonic PBS and normal PBS, anti-rabbit secondary antibody (1:1000; Alexa Fluor 594, Life Technologies) mixed with Alexa Fluor 488-coupled phalloidin (1: 500; Life Technologies) and 4,6-diamidino-2-phenylindole (1:500; DAPI, Sigma-Aldrich) were applied and incubated for 1 h at room temperature. After the previous washing protocol was repeated, the sections were mounted with Dako mounting medium (Dako, USA) and analyzed either with a Leica DM 5500B fluorescence microscope or with the CLSM Leica Stellaris 5 upright confocal microscope, provided by the Center for Microscopy and Image Analysis of the University of Zurich, using the software LAS X with a magnification of × 63 and a step size of 0.30 μm. The images obtained with the confocal microscope were processed with the Bitplane Imaris 10.1.1 software.

### RNA isolation and quantitative real-time PCR

Total RNA from kidney and jejunum samples was extracted by homogenizing the tissue in TRIzol (Invitrogen, USA) using MagNa Lyser Green Beads (Roche, Switzerland) and the Precellys 24 homogenizer (Bertin Instruments), followed by chloroform extraction. Total RNA was purified with the NucleoSpin RNA mini kit (Macherey–Nagel) in accordance with the manufacturer’s protocol and the purity measured with the ND-1000 NanoDrop spectrophotometer (Thermo Scientific, USA). cDNA was transcribed according to the TaqMan Reverse Transcription protocol (Thermo Scientific, USA), by incubating RNA (10 min at 25 °C, 30 min at 48 °C, 5 min at 95 °C, and holding at 4 °C) in the presence and absence of reverse transcriptase, the latter samples serving as negative control. Gene expression was determined by qRT-PCR incubating cDNAs with the KAPA Probe Fast qPCR Universal Master Mix (2x) Kit (Kapa Biosystems) for probe-based qPCR or the PowerUp SYBR Green Master Mix (Applied Biosystems) for dye-based reactions. Gene expression was determined with the QuantStudio 6 Pro Real-Time PCR System (Applied Biosystems), with the following qRT-PCR conditions: 95 °C for 20 s, 40 cycles of 95 °C for 3 s and 60 °C for 30 s. Gene expression levels were normalized to that of HPRT or 18S rRNA (Applied Biosystems (REF 4310893E)) and calculated with the 2^−dCT^ method. The used primers and FAM/TAMRA labeled probes (when applicable) are listed in Supplementary Table 1.

### Reverse transcription PCR (RT-PCR)

Intestinal and renal cDNA obtained from total RNA as described above was used as template to perform PCR. Primers for *Cldn3* were designed with the help of National Center of Biotechnology Information (NCBI), checked for specificity by BLAST and ordered from Microsynth (Balgach, Switzerland; Supplementary Table 1). The REDTaq ReadyMix PCR reaction mix (Sigma-Aldrich, REF R2523-100RXN) containing Taq DNA Polymerase was mixed with primers and cDNA, and the PCR (94 °C for 120 s followed by 35 cycles of 94 °C for 30 s, 60 °C for 30 s, and 72 °C for 30 s and final extension of 72 °C for 300 s) was performed on the thermocycler Techne TC-512 (Barloworld Scientific, Ltd., UK). The PCR products were then run on a 2% agarose gel in the presence of GelRed Nucleic Acid Stain (Biotium, REF 41003) and the amplicons visualized under UV light with the Infinity BioVision image acquisition software (Vilber, France).

### Statistical analysis

All results were analyzed with the GraphPad Prism 10.1.2 software, and the data is presented as single values together with the means ± standard deviation. Outlier data points were determined with Grubbs’ test. Outliers for any urinary parameter were excluded in further urinary comparisons. In case of two groups, normal distribution was confirmed by the Shapiro–Wilk test and statistical significances were calculated by an unpaired *t*-test. For analysis of more than two groups, two-way ANOVA followed by Tukey’s multiple comparisons test was used. *P* values < 0.05 were considered significant.

## Results

### Cldn3 mRNA is expressed along the whole intestinal tract in mice

RT-PCR on RNA extracted from intestinal mucosa scrapings of WT mice detected *Cldn3* mRNA along the whole intestinal tract (Supplementary Fig. 1A). The lack of genomic contamination was confirmed by the near absence of amplicons in PCR using as template RNA samples that were not subjected to reverse transcription (data not shown). Likewise, immunohistochemistry demonstrated the presence of Cldn3 protein in enterocytes (Supplementary Fig. 1B).

### *Deletion of Cldn3 does not alter basal levels of fecal, urinary, and plasma Pi and Ca*^*2*+^*but increases calcitriol concentration*

**Fig. 1:**
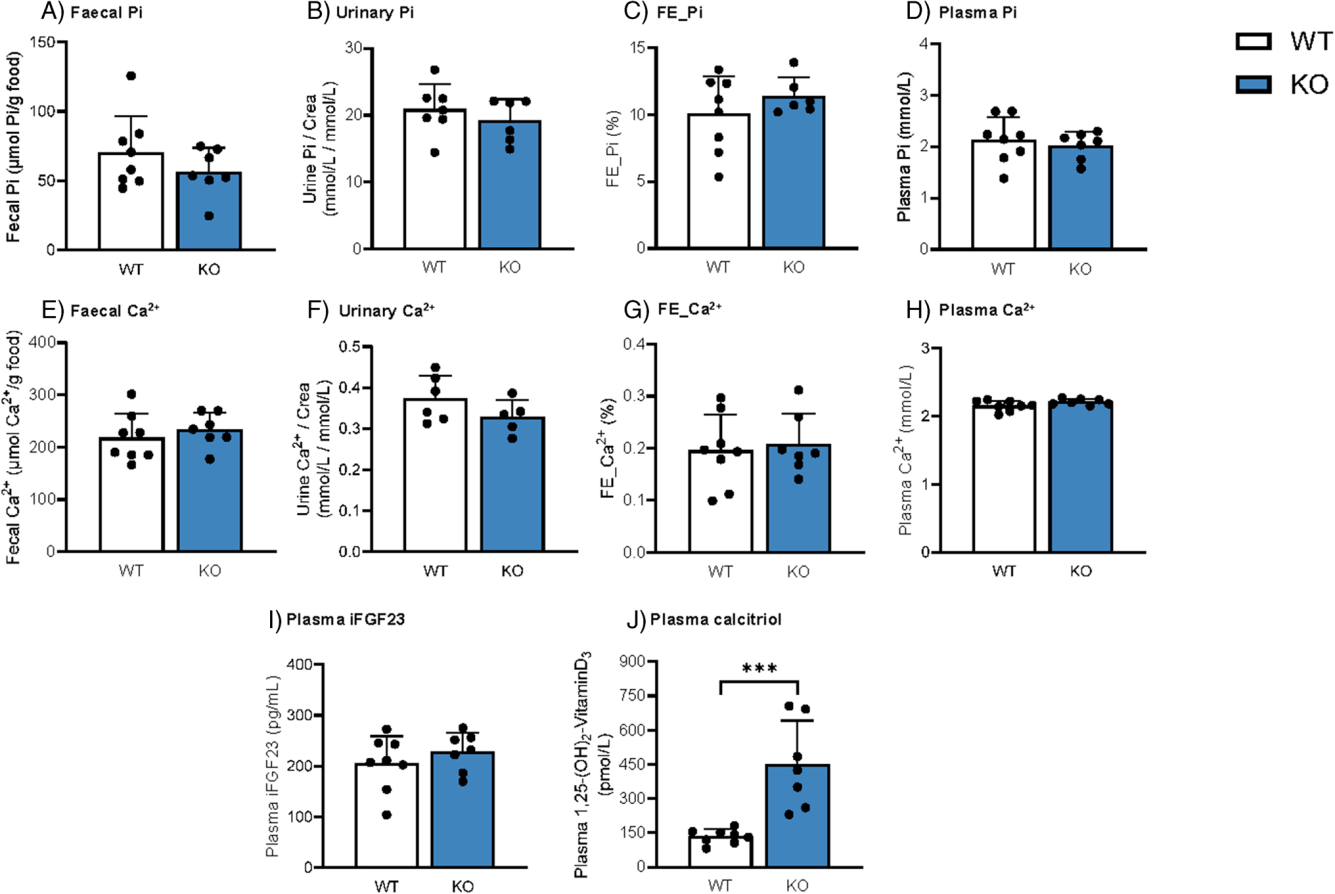
Deletion of *Cldn3* does not alter basal levels of fecal, urinary, and plasma Pi and Ca^2+^ but increases calcitriol concentration. **A**, **E** Fecal excretions of Pi and Ca^2+^ normalized to food intake. **B**, **F** Urinary Pi and Ca^2+^ corrected by creatinine (crea) excretion. **C**, **G** Renal fractional excretion (FE %) of Pi and Ca^2+^. **D**, **H** Plasma Pi and Ca.^2+^ concentrations. **I**, **J** Plasma concentrations of intact FGF23 (iFGF23) and calcitriol. *N* = 7–8 mice per group. **A**–**J** Unpaired *t*-test. ***P < 0.001

In a first series of experiments, we aimed to elucidate whether global *Cldn3* KO mice exhibit a baseline phenotype of altered Pi handling and homeostasis by analyzing several Pi-related parameters in mice fed on standard diet. Fecal and urinary excretion of Pi (the latter either corrected by creatinine or calculated as fractional excretion (FE)) as well as plasma Pi was similar in WT and *Cldn3* KO littermates (Fig. 1A–D), as were total Ca^2+^ levels (Fig. 1E–H). Na^+^, Cl^−^, and K^+^ concentrations were not different between genotypes in neither plasma nor urine (Supplementary Fig. 2A-I). Although the urinary excretion of Mg^2+^ was slightly elevated in the KO animals when corrected by creatinine, this difference disappeared upon the calculation of the FE (Supplementary Fig. 2 J-K). Plasma levels of Mg^2+^ were similar in WT and *Cldn3* KO mice (Supplementary Fig. 2L).

The plasma levels of the phosphaturic hormone FGF23 were similar in both genotypes in baseline conditions (Fig. 1I). However, plasma calcitriol, whose levels stimulate intestinal absorption of Pi as well as intestinal and renal transport of Ca^2+^, was significantly elevated in *Cldn3* KO animals (Fig. 1J), despite the absence of differences in fecal and urinary Pi and Ca^2+^ excretion between the groups.

### *High Pi diet does not alter Pi levels, but reduces urinary excretion of Ca*^*2*+^*and further increases calcitriol concentration in Cldn3 KO*

**Fig. 2:**
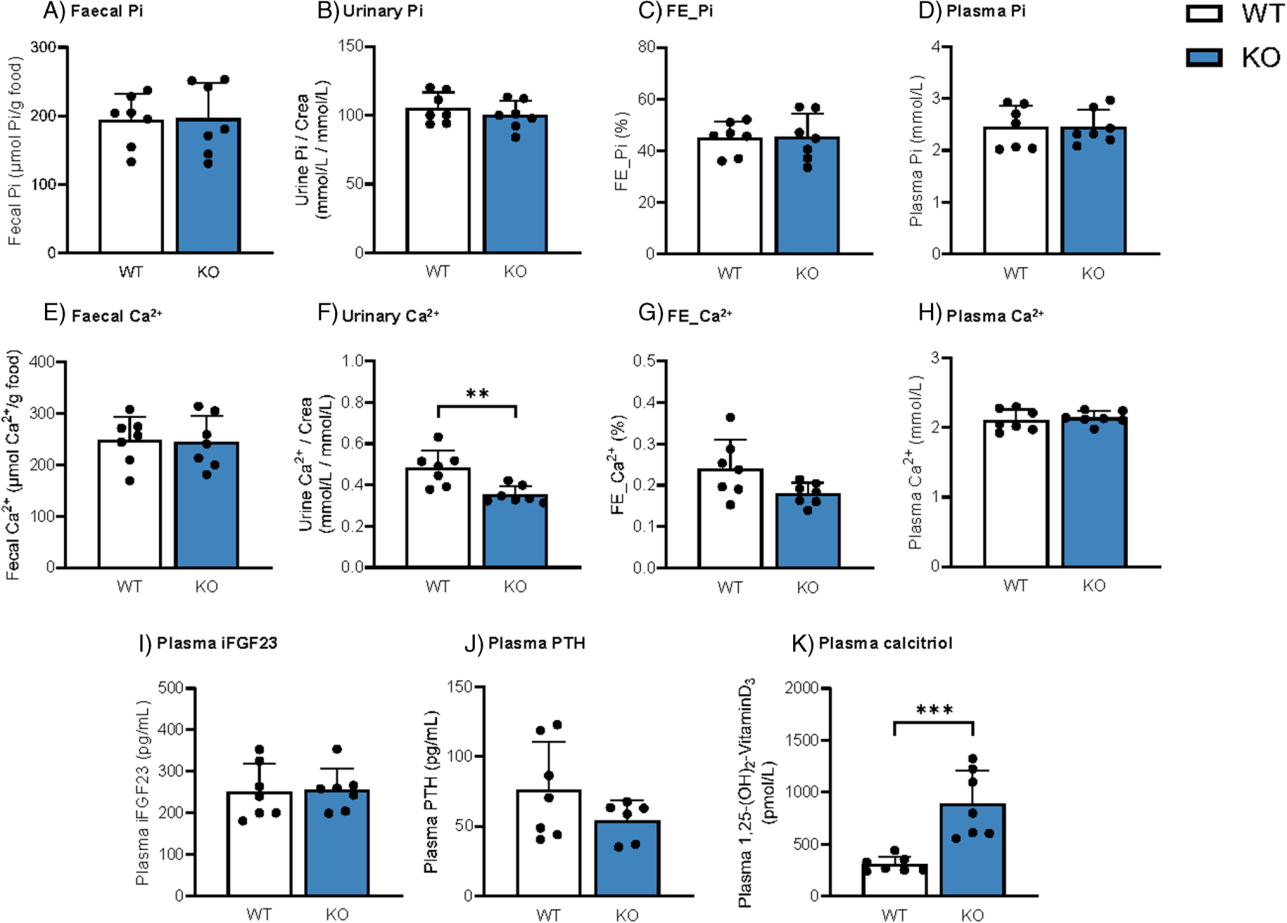
Deletion of *Cldn3* does not alter Pi levels but reduces urinary excretion of Ca^2+^ and further increases calcitriol concentration in mice on high Pi diet. **A**, **E** Fecal excretions of Pi and Ca^2+^ normalized to food intake. **B**, **F** Urinary Pi and Ca^2+^ corrected by creatinine (crea) excretion. **C**, **G** Renal fractional excretion (FE %) of Pi and Ca^2+^. **D**, **H** Plasma Pi and Ca.^2+^ concentrations. **I**–**K** Plasma concentrations of intact FGF23 (iFGF23), parathyroid hormone (PTH), and calcitriol, respectively. *N* = 7–8 mice per group. **A**–**K** Unpaired *t*-test. **P < 0.01 and ***P < 0.001

In a second set of experiments, WT and *Cldn3* KO mice were placed on high Pi diet (HP) for 3 days to increase intestinal paracellular Pi absorption. Fecal and urinary output of Pi, as well as plasma Pi, was similar in both genotypes (Fig. 2A–D). As expected, fecal and urinary excretion of Pi reflected the dietary content, and both were higher in mice fed on HP than in those fed on normal diet (compare Figs. 1A–C with 2A–C, though determinations were done independently for each diet). Fecal excretion and plasma levels of Ca^2+^ were also similar in WT and *Cldn3* KOs, while the urinary excretion of Ca^2+^ was lower in KO mice than in their WT littermates, a difference more evident when excretion was normalized to creatinine (Fig. 2E–H). Na^+^, Cl^−^, K^+^, and Mg^2+^ levels were similar between genotypes in both plasma and urine (Supplementary Fig. 3A-L).

Upon 3 days of HP, no differences between WT and *Cldn3* KO littermates were observed in the plasma levels of intact FGF23 (Fig. 2I) or PTH (Fig. 2J). On the other hand, calcitriol levels remained highly elevated in the *Cldn3* KO group (Fig. 2K). The persistent elevation of calcitriol levels under 3 days of HP may reflect the impact of high phosphate on intestinal calcium availability.

### Deletion of Cldn3 does not alter intestinal fluxes of Pi

**Fig. 3:**
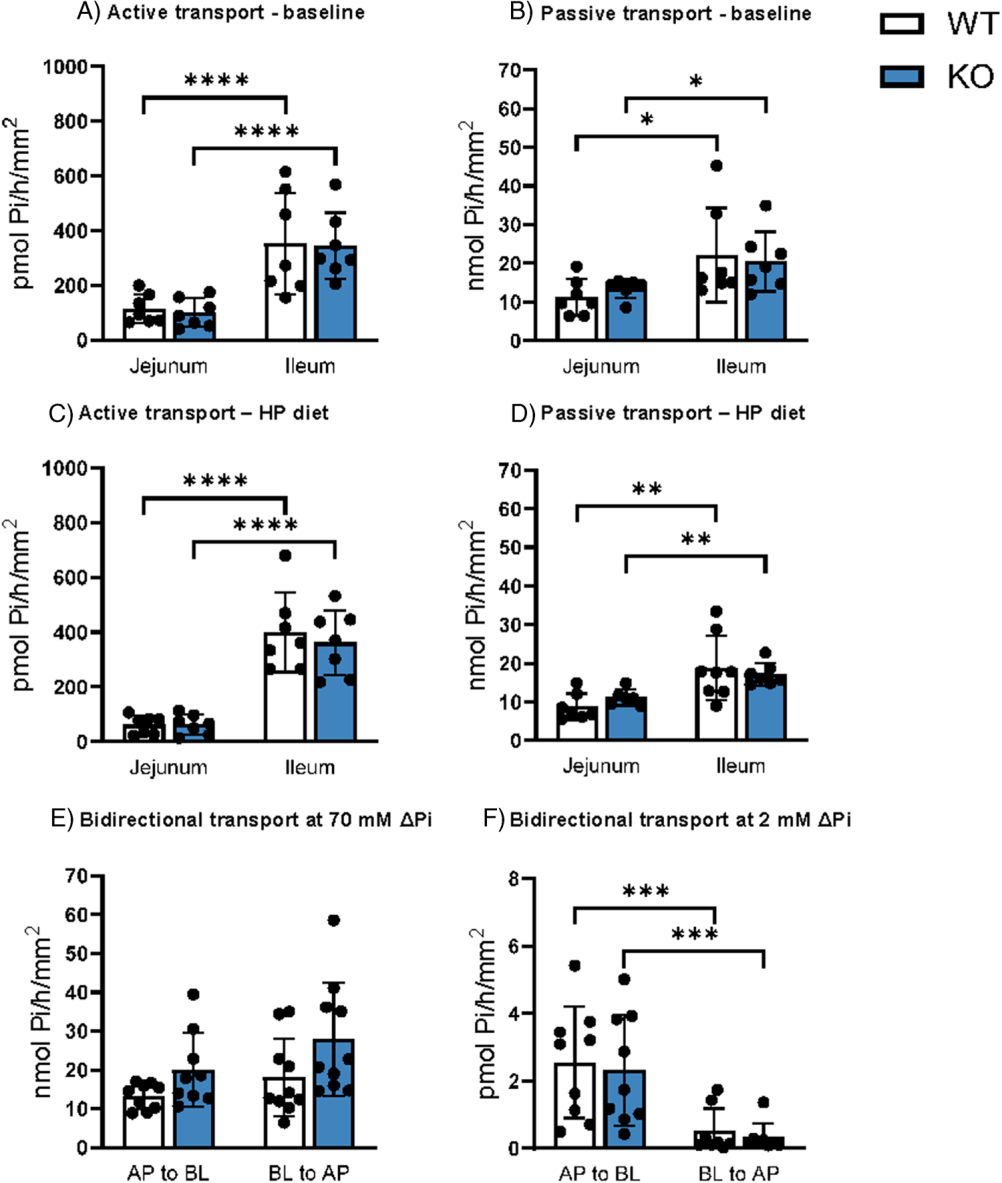
Deletion of *Cldn3* does not alter intestinal fluxes of Pi. ^32^P fluxes in the jejunum and ileum of WT and *Cldn3* KO mice were measured with the Ussing chamber technique. **A**, **B** Active and passive transports under baseline dietary conditions. **C**, **D** Active and passive transports upon high dietary Pi challenge. **E**, **F** Jejunal transport values upon imposing 70 mM and 2 mM apical-to-basolateral (AP to BL) and basolateral-to-apical (BL to AP) Pi gradients. *N* = 7–10 mice per group. **A**–**F** Two-way ANOVA followed by Tukey’s multiple comparisons test. *P < 0.05, **P < 0.01, ***P < 0.001, and ****P < 0.0001

To directly address the effect of *Cldn3* deletion on intestinal transport of Pi, we measured ^32^P fluxes across samples of jejunum and ileum in Ussing chambers. As reported previously [20], fluxes were measured in the presence of two different apical-to-basolateral gradients of Pi that allow to discriminate between active and passive transport mechanisms: a 100 µM gradient (100 µM apical Pi with no Pi added to the basolateral chamber) favors the detection of active transport whereas a 70 mM gradient (70 mM apical Pi with no Pi added to the basolateral chamber) favors detection of the passive process. As expected, in both genotypes and dietary conditions, active transport was higher in the ileum as compared with the jejunum (Fig. 3A, C), reflecting the pattern of expression of NaPi-IIb. However, we found no differences on this active component between genotypes. Passive transport also tended to be higher in the ileum than the jejunum, but again no differences were detected between WT and *Cldn3* KO mice neither under normal dietary conditions (Fig. 3B) nor upon 3 days with high dietary Pi (Fig. 3D).

The bidirectional nature of the passive component was confirmed in jejunal samples from mice fed on standard chow by imposing a 70 mM Pi gradient both apical-to-basolateral (Pi added to the apical chamber and media sampled from the basolateral compartment) as well as basolateral-to-apical (Pi added to the basolateral chamber and media sampled from the apical compartment). As expected, the magnitude of transport was similar for both gradients, with no differences detected between genotypes either (Fig. 3E). Similar experiments were also performed imposing a 2 mM Pi gradient (Fig. 3F). Interestingly, in this experiment, the transport driven by the apical-to-basolateral gradient was significantly larger than the transport elicited by the basolateral-to-apical gradient (though far smaller than the one detected with the 70 mM gradients).

### Deletion of Cldn3 does not alter absolute and relative transepithelial permeabilities for Pi, Ca^2+^, Na^+^, and Cl^−^

**Fig. 4:**
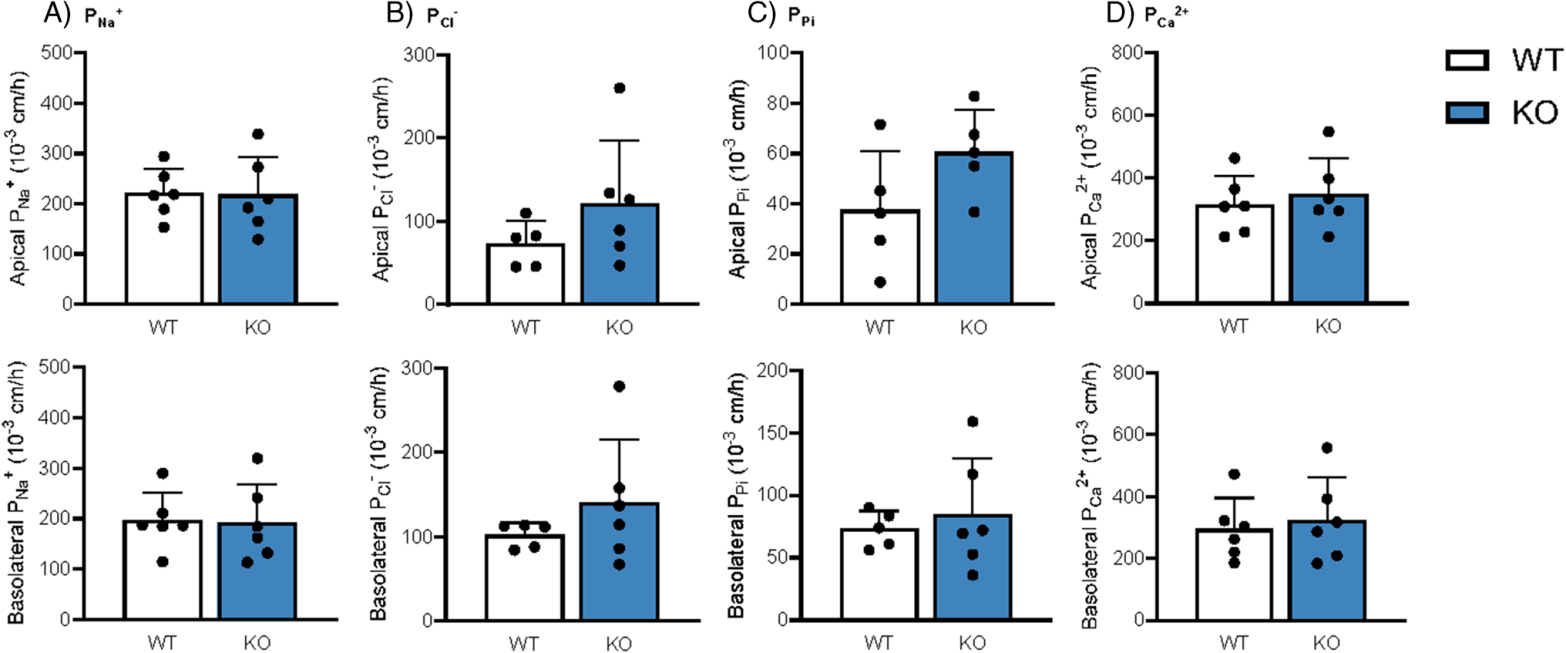
Deletion of *Cldn3* does not alter absolute permeabilities for Pi, Ca^2+^, Na^+^, and Cl^−^ in the jejunum of mice fed on high Pi. Ion permeabilities were calculated from diffusion potential and transepithelial resistance measured with the electrical Ussing chamber technique. Absolute permeabilities for **A** Na^+^, **B** Cl^−^, **C** Pi, and **D** Ca^2+^ are shown for apical (top) and basolateral (bottom) solution exchange, respectively. *N* = 6 mice per group. **A**–**D** Unpaired *t*-test

**Fig. 5:**
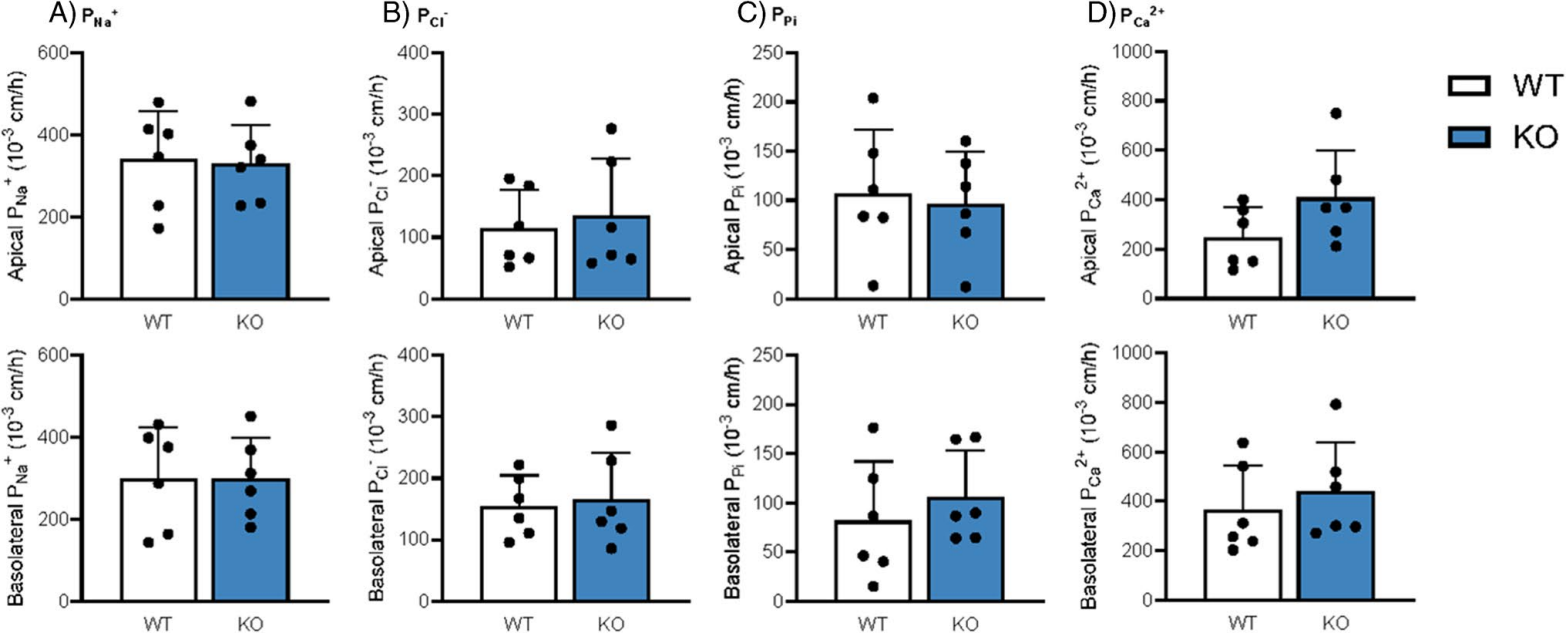
Deletion of *Cldn3* does not alter absolute permeabilities for Pi, Ca^2+^, Na^+^, and Cl^−^ in the ileum of mice fed on high Pi. Ion permeabilities were calculated from diffusion potential and transepithelial resistance measured with the electrical Ussing chamber technique. Absolute permeabilities for **A** Na^+^, **B** Cl^−^, **C** Pi, and **D** Ca^2+^ are shown for apical (top) and basolateral (bottom) solution exchange, respectively. *N* = 6 mice per group. **A**–**D** Unpaired *t*-test

Since *Cldn3* KO mice showed diminished urinary Ca^2+^ excretion upon the high dietary Pi challenge, we next directly assessed the intestinal permeability of WT and *Cldn3* KO mice fed 3 days on HP diet. Ileal and jejunal permeabilities for Na^+^, Cl^−^, Pi, and Ca^2+^ were examined with the electrical Ussing chamber technique. No differences were found in the permeabilities for Na^+^, Cl^−^, and Pi in the jejunum or ileum (Figs. 4A–C and 5A–C), in line with their normal urinary and fecal excretion values. Despite the reduction observed in urinary Ca^2+^ excretion, the intestinal permeability for Ca^2+^ was not affected by the genetic deletion of *Cldn3* either (Figs. 4D and 5D). The relative permeabilities of the analyzed ions were also similar in WT and *Cldn3* KO mice both in the jejunum (Supplementary Fig. 4A-D) and ileum (Supplementary Fig. 5A-D).

### Deletion of Cldn3 does not alter intestinal expression of claudin-7 and of NaPi-IIb

**Fig. 6:**
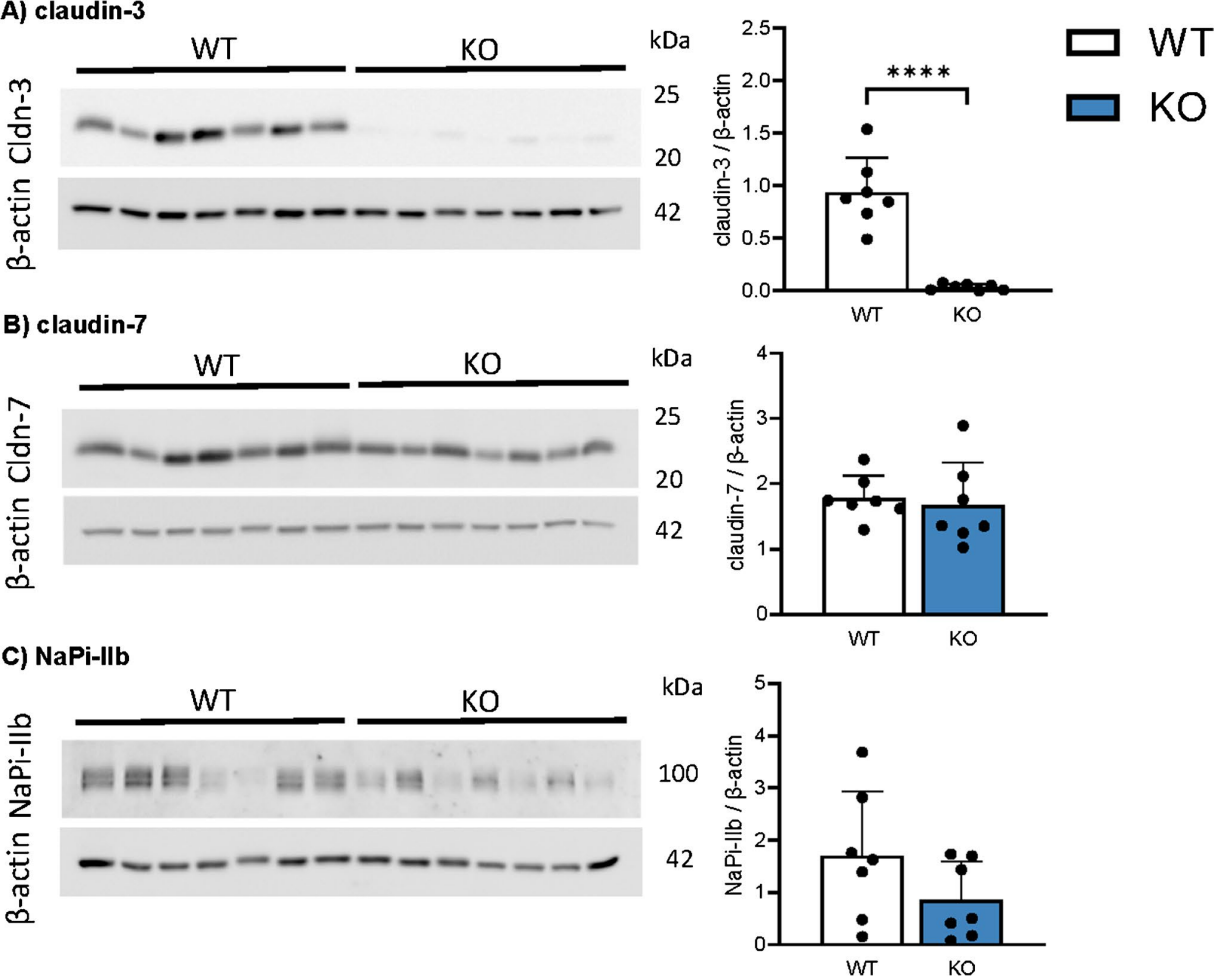
Deletion of *Cldn3* does not alter the basal expression of claudin-7 and of NaPi-IIb in the ileum. The protein expression of **A** claudin-3, **B** claudin-7, and **C** NaPi-IIb was analyzed by Western blotting in the ileum of mice fed on standard diet. Graphs show expression of analyzed proteins corrected to the abundance of β-actin. *N* = 7 mice per group. **A**–**C** Unpaired *t*-test. ****P < 0.0001

**Fig. 7:**
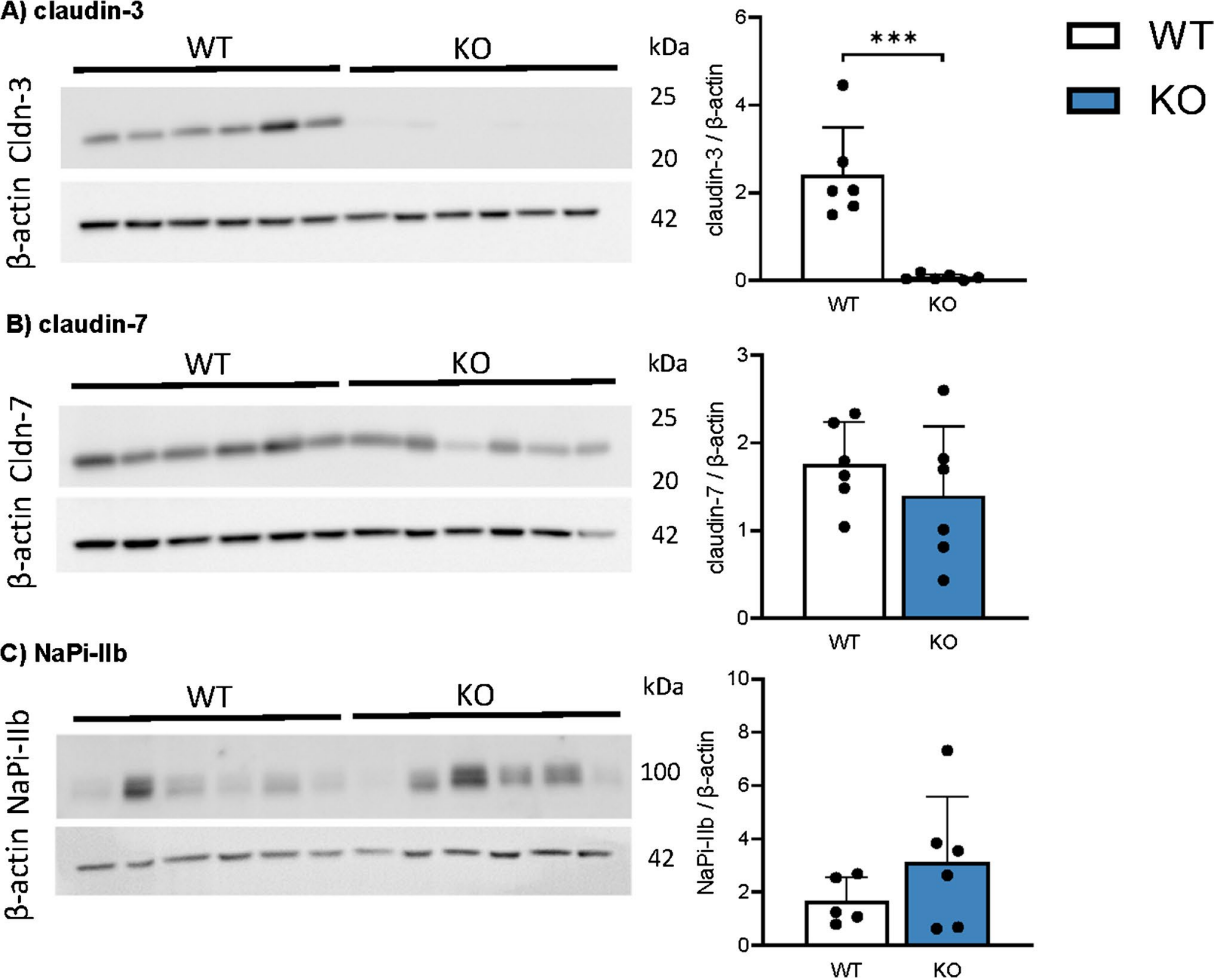
Deletion of *Cldn3* does not alter the expression of claudin-7 and of NaPi-IIb in the ileum of mice fed on high Pi. The protein expression of **A** claudin-3, **B** claudin-7, and **C** NaPi-IIb was analyzed by Western blotting in the ileum of mice fed on high Pi. Graphs show expression of analyzed proteins corrected to the abundance of β-actin. *N* = 6 mice per group. **A**–**C** Unpaired *t*-test. ***P < 0.001

**Fig. 8:**
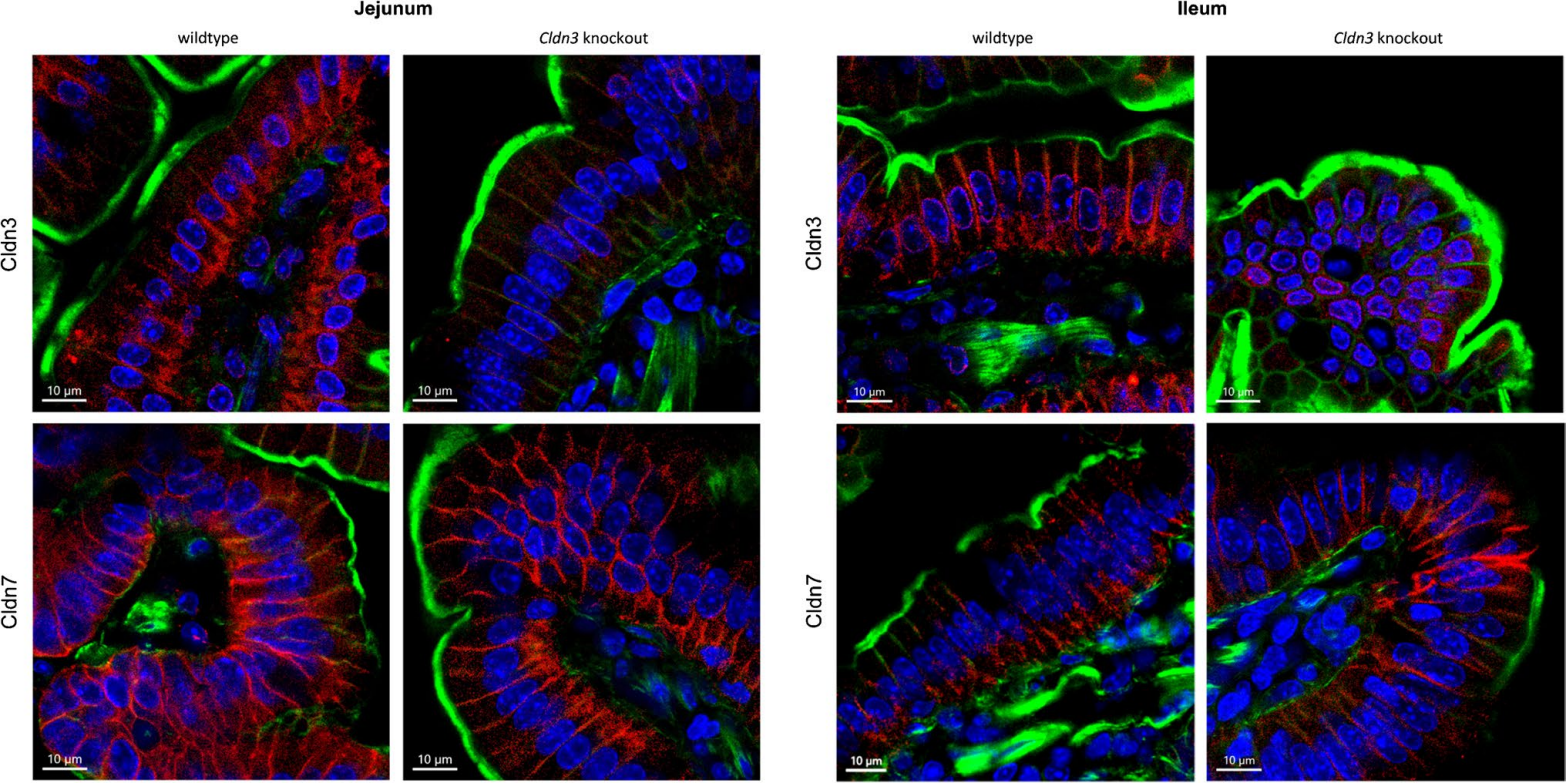
Deletion of *Cldn3* does not alter the subcellular localization of claudin-7. Immunofluorescence of intestinal sections of wild-type and *Cldn3* KO mice using a Cldn3 or Cldn7 antibody (red), phalloidin (to stain actin: green), and DAPI (blue). Representative images in the jejunum and ileum are shown. Magnification of × 63, scale bar: 10 µm. Larger areas of the same sections are shown in Supplementary Fig. 7

As expected, the protein expression of Cldn3 was almost absent from ileum from *Cldn3* KO mice compared with tissue from WT, both in animals fed on standard chow (Fig. 6A) and in those fed on high Pi (Fig. 7A). This near absence of Cldn3 was also confirmed by immunofluorescence: Cldn3 antibodies detected a signal located along the lateral membrane of enterocytes in ileum in WT mice, while this signal was absent in tissue from *Cldn3* KO mice (Fig. 8 and Supplementary Figs. 1B and 7). Similar observations were made in the duodenum, jejunum, and proximal colon. Moreover, *Cldn3* deletion did not alter the ileal protein abundance of claudin-7, another claudin potentially involved in paracellular transport of ions (Figs. 6B and 7B). Unfortunately, commercial antibodies failed to detect specific bands for claudin-7 and claudin-8, the presence of the latter is required for proper expression of claudin-4 [17]. Similar to claudin-7, the protein expression of NaPi-IIb was unchanged in the ileum of WT and *Cldn3* KO fed both on standard (Fig. 6C) and high Pi chow (Fig. 7C).

The absence of *Cldn3* was also confirmed in jejunal samples from *Cldn3* KO mice fed on HP (Supplementary Fig. 6A). The protein abundance of claudin-7 and NaPi-IIb in *Cldn3* KO remained similar to WT in this intestinal segment as well (Supplementary Fig. 6B-C).

To assess whether the deletion of *Cldn3* altered the intestinal localization of additional claudin subtypes, specifically of claudin-7, confocal microscopy images were obtained in the jejunum and ileum of WT and *Cldn3* KO mice (Fig. 8 and Supplementary Fig. 7). Both claudin-3 and claudin-7 showed localization to the lateral membrane of intestinal cells, with the signal for claudin-3 essentially depleted in KO animals. The subcellular localization of claudin-7 did not seem to be affected by the deletion of the *Cldn3* gene in neither the jejunal nor the ileal segment.

### Deletion of Cldn3 does not alter renal expression of NaPi-IIa

Deletion of *Cldn3* did not affect the renal expression of NaPi-IIa, the cotransporter chiefly controlling renal reabsorption of Pi, the abundance of which was similar in WT and KO mice fed both standard and high Pi diets (Supplementary Fig. 8A, B).

### *Deletion of Cldn3 does not alter intestinal protein expression of several regulators of Ca*^*2*+^*absorption*

**Fig. 9:**
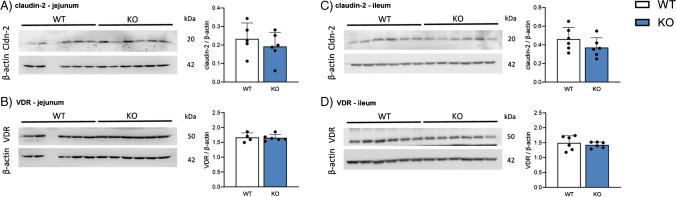
Deletion of *Cldn3* does not alter intestinal protein expression of several regulators of Ca^2+^ absorption. The protein expression of **A**, **C** claudin-2 and **B**, **D** of the vitamin D receptor (VDR) was analyzed by Western blotting in the jejunum and ileum of mice fed on high Pi. Graphs show expression of analyzed proteins corrected to the abundance of β-actin. *N* = 6 mice per group. **A**–**D** Unpaired *t*-test

We next analyzed the protein expression of several targets involved in intestinal Ca^2+^ handling. In the jejunum of mice fed on HP, claudin-2, a tight junction protein involved in intestinal Ca^2+^ absorption (and renal proximal tubular Ca^2+^ reabsorption) showed similar expression in WT and *Cldn3* KO mice (Fig. 9A), as did the jejunal expression of the vitamin D receptor (VDR; Fig. 9B). The same results were observed in the ileum (Fig. 9C, D). Calbindin-D28k expression also showed similar values in both genotypes in the jejunum as well as in the ileum (Supplementary Fig. 9A, B).

### Deletion of Cldn3 results in elevated renal mRNA expression of Cyp27b1

**Fig. 10:**
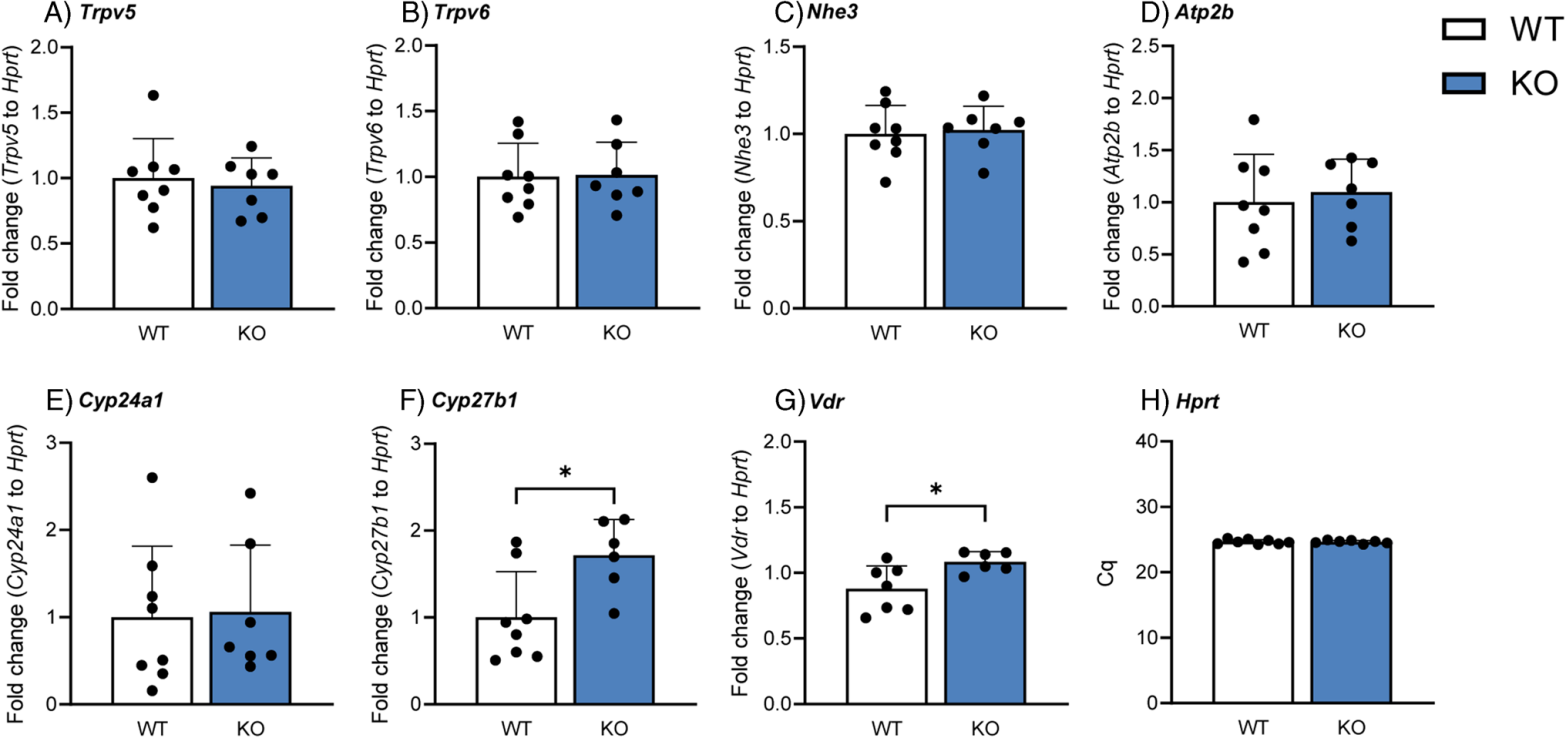
Deletion of *Cldn3* results in elevated renal mRNA expression of *Cyp27b1*. Renal gene expression of **A** *Trpv5*, **B** *Trpv6*, **C** *Nhe3*, **D** *Atp2b*, **E** *Cyp24a1*, **F** *Cyp27b1*, **G** *vitamin D receptor (VDR)*, and **H** *Hprt* in mice fed on standard diet. *N* = 7–8 mice per group. **A**–**H** Unpaired *t*-test. *P < 0.05

Next, we assessed the mRNA expression of several genes related to Ca^2+^ handling in the kidneys of WT and *Cldn3* KO mice. In agreement with the absence of differences between genotypes in urinary and plasma Ca^2+^ levels under baseline conditions, the gene expression of the epithelial Ca^2+^ channels *Trpv5* and *Trpv6* (Fig. 10 A, B) and the Na^+^/H^+^ exchanger 3 (*Nhe3*, Fig. 10C), whose activity drives the passive reabsorption of approximately 60–70% of all filtered Ca^2+^ [39], remained unaffected. Additionally, the gene expression of the plasma membrane Ca^2+^ ATPase (*Atp2b*, Fig. 10D) was also similar in both groups, as was *Cyp24a1* (Fig. 10E), the gene coding for the enzyme responsible for the 24-hydroxylation and subsequent degradation of the active form of vitamin D_3_. However, the expression of *Cyp27b1*, the gene encoding the enzyme responsible for the last hydroxylation step in the generation of active vitamin D_3_, as well as the *Vdr* was increased in *Cldn3* KO mice (Fig. 10F, G). The housekeeping gene *Hprt* was not affected by the genetic deletion of *Cldn3* under baseline conditions (Fig. 10H).

**Fig. 11:**
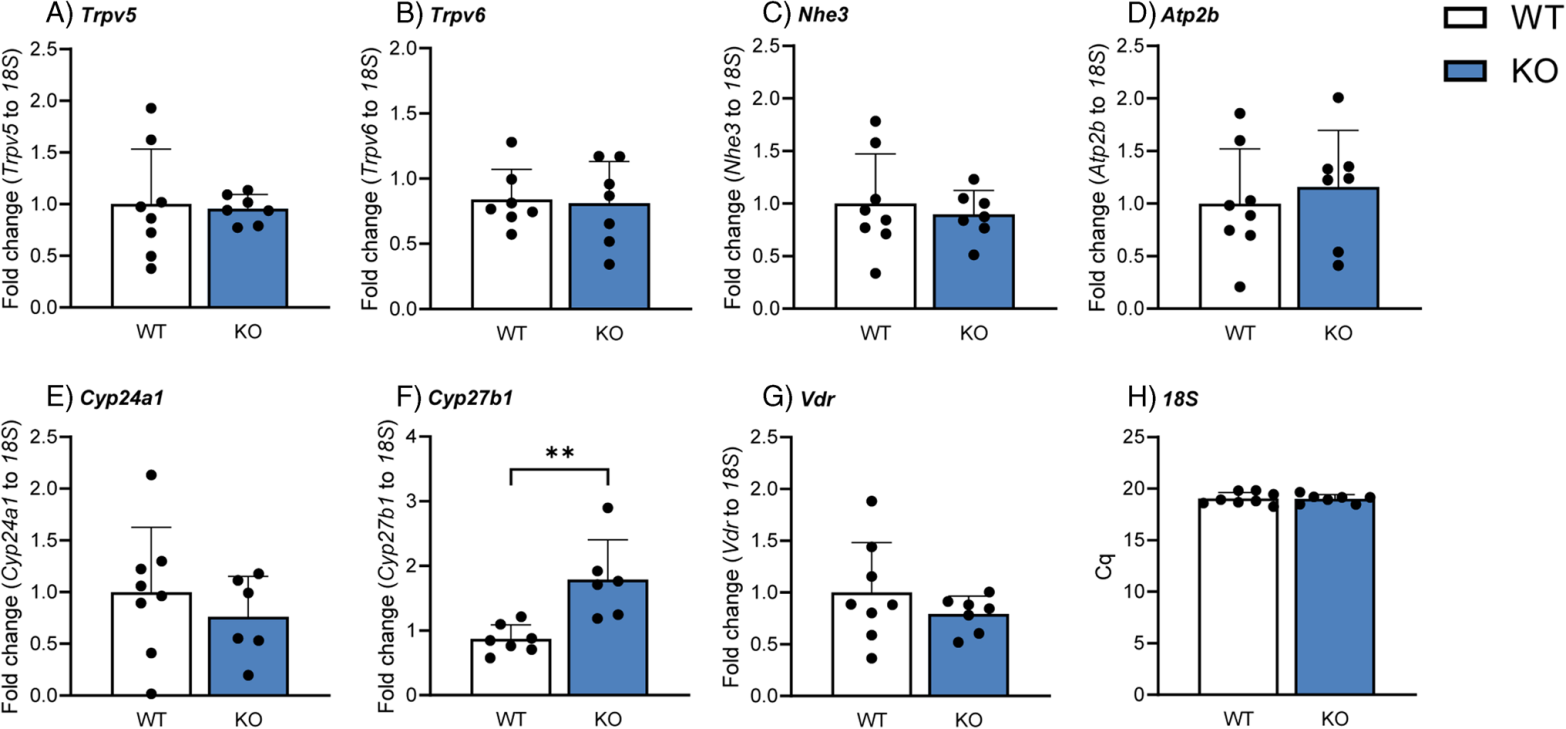
Deletion of *Cldn3* results in elevated renal mRNA expression of *Cyp27b1* in mice fed on high Pi. Renal gene expression of **A** *Trpv5*, **B** *Trpv6*, **C** *Nhe3*, **D** *Atp2b*, **E** *Cyp24a1*, **F** *Cyp27b1*, **G** *vitamin D receptor (VDR)*, and **H** *18S* in mice fed on high Pi. *N* = 7–8 mice per group. **A**–**H** Unpaired *t*-test. **P < 0.01

Upon 3 days of high Pi dietary challenge, no alterations in renal gene expression were observed for *Trpv5*, *Trpv6*, *Nhe3*, *Atp2b*, or *Cyp24a1* (Fig. 11A–E). Renal *Cyp27b1* mRNA levels (Fig. 11F) were significantly higher in *Cldn3* KO animals, in line with the elevated plasma calcitriol in this group. Unlike under baseline conditions, *Vdr* mRNA levels were not affected by the deletion of *Cldn3* in mice challenged with HP (Fig. 11G). Since *Hprt* gene expression tended to be higher in the KO mice upon high Pi diet (data not shown), mRNA levels of the aforementioned targets were normalized to that of *18S*, whose gene expression remained similar in both genotypes (Fig. 11H).

The gene expression of the mentioned candidates was also measured in the jejunum. While *Trpv5* and *Trpv6* transcripts were not detected in this intestinal segment, *Nhe3*, *Atp2b*, and *Vdr* showed similar expression in WT and *Cldn3* KO mice both under baseline conditions (Supplementary Fig. 10A-C), as well as upon high Pi dietary challenge (Supplementary Fig. 10F-H)*.* Moreover, *Cldn12*, a constituent of tight junctions associated with Ca^2+^ absorption, was present in the jejunum, but its levels did not show differences between genotypes (Supplementary Fig. 10D, I).

## Discussion

While the renal control of Pi homeostasis has been thoroughly investigated, much less is known about the intestinal absorption of this anion. This is especially true for the paracellular pathway, mediated by tight junctions, which is the mode of transport likely contributing to the majority of intestinal Pi absorption in humans consuming a “Western diet” [13, 30]. The understanding of the molecular identity of this pathway is therefore of great importance, particularly in conditions associated with renal failure (such as CKD), when the intestinal absorption is the only remaining means to control serum Pi levels [32]. Claudin-3, a tight junction protein expressed in a variety of epithelia such as the gastrointestinal tract and the kidney, has been recently proposed to have a Pi sealing function in epithelia [4, 26]. Here, we assessed the phenotype of global *Cldn3* KO mice, in which intestinal deletion of the protein was confirmed by immunofluorescence and immunoblotting, with regard to Pi homeostasis. Because Pi and Ca^2+^ share common hormonal regulators (calcitriol, PTH, and FGF23), Ca^2+^ homeostasis was analyzed as well. Determinations were performed under normal dietary conditions, to assess the basal phenotype, as well as upon 3 days of high dietary Pi challenge. High dietary Pi imposes a larger gradient of Pi between the intestinal lumen and blood/interstitium increasing possible differences between WT mice (with intestinal tight junctions sealed for Pi) and *Cldn3* KO (whose tight junctions would be leakier for Pi if claudin-3 indeed seals the paracellular pore for the anion).

Our experiments lead to several important findings. Firstly, the genetic deletion of *Cldn3* did not lead to compensation in intestinal protein abundance and/or localization by other claudin subtypes, namely, claudin-4 and claudin-7, despite previous reports noting the ability of claudins to compensate for the other’s absence [9, 38]. The basal phenotype of the *Cldn3* KO animals did not reveal any changes of Pi and Ca^2+^ values in urine, feces, or plasma. However, plasma calcitriol levels were elevated in these mice, probably due to higher renal synthesis, as suggested by the higher mRNA expression of *Cyp27b1*. This is a surprising finding in face of the normal Pi and Ca^2+^ concentrations in plasma and urine. On the other hand, FGF23 showed similar levels in both genotypes, despite calcitriol stimulating the production and release of FGF23 by osteocytes [22, 31]. The elevation of calcitriol in *Cldn3* KO mice persisted also after 3 days of high Pi feeding, although FGF23, PTH (whose secretion is negatively regulated by calcitriol [29, 35]), and Pi levels remained unaffected. Under both dietary conditions, no differences were found in the renal protein expression of NaPi-IIa, the main Na^+^/Pi cotransporter in the BBM of proximal tubules, in line with the similar urinary excretion of Pi and normal levels of phosphaturic hormones.

Our group has previously shown that the entire axis of the mouse intestine has a high paracellular permeability for Pi, with the ileum showing the highest permeability [20]. Therefore, we chose this segment to compare active and passive ^32^P fluxes in WT and *Cldn3* KO mice. Additionally, fluxes were also measured in the jejunum, as in mice the expression of NaPi-IIb is by far smaller in this segment than in the ileum [27]; therefore, the contribution of the transcellular pathway to overall Pi absorption is likely small in the jejunum and maximal in the ileum. Accordingly, under both dietary conditions, the active ^32^P transport measured in the jejunum was much lower than that in the ileum. However, this active component, as well as ileal and jejunal NaPi-IIb protein expression, was similar in *Cldn3* KO and WT mice despite the well-established stimulatory effect of calcitriol on NaPi-IIb expression as well as on the active transport of Pi [12, 15]. Under both dietary conditions, the paracellular flux of Pi was also higher in the ileum than in the jejunum (corresponding to the highest Pi permeability reported in the ileal segment [20]), but it was not altered by the absence of *Cldn3*. The symmetry of the transport (a hallmark of passive transport) was confirmed by measuring equivalent fluxes and permeabilities when establishing a 70 mM Pi concentration gradient from either side of the epithelium. Notably, a 2 mM Pi concentration gradient did not result in similar fluxes but led to higher apical-to-basolateral than basolateral-to-apical transport, suggesting that this gradient is not large enough to drive sufficient passive transport as to mask the contribution of the active component. In contrast to the paracellular pathway, transcellular absorption of Pi is unidirectional (apical-to-basolateral) and is mostly driven by NaPi-IIb whose affinity for Pi is very high (Km in the µM range [16]). This high affinity together with the use of a relatively small Pi gradient (for comparison, up to ≈20 mM Pi difference between intestinal lumen and plasma has been reported in mice under normal dietary conditions [20, 25]) may have contributed to the detection of mostly the active component in the bidirectional experiments performed with the 2 mM gradient. The observation that deletion of *Cldn3* did not alter intestinal tight junction permeability for Pi was subsequently confirmed with dilution potential measurements in the jejunum and ileum of mice fed on high Pi, where the permeability of the epithelia for Pi (as well as for Na^+^, Ca^2+^, and Cl^−^) was found to be similar in *Cldn3* KO and WT animals. These findings are in contrast with the previous report suggesting that *Cldn3* tightens the paracellular route for Pi absorption [11]. This suggestion was exclusively based on the observation that transport of Pi into everted sacs from the jejunum, ileum, and colon from *Cldn3* KO was higher than in segments from WT mice. These experiments were performed in the presence of 1.2 mM Pi (a concentration that as discussed above does not effectively discriminate between the active and passive components), but with no Na^+^ added to the transport solution (which may have reduced the Na^+^-dependent component). The mice used in Hashimoto’s work as well as the ones used in ours were both generated on a mixed background (129S and C57BL/6 backcrossed into C57BL/6). In both lines, the single exon of *Cldn3* is deleted; therefore, the genetic background of the animals is not likely to explain the differences between studies.

The permeability for Ca^2+^ in the jejunum and ileum of *Cldn3* KO mice was similar to the one observed in WT upon high Pi challenge. Moreover, none of the analyzed channels/transporters associated with Ca^2+^ handling was altered in the intestine of KO mice. For instance, the jejunal and ileal protein expression of Cldn2 and jejunal gene expression of *Cldn12* remained unaffected in *Cldn3* KOs, despite the stimulating effect of calcitriol on these genes described both in vivo and in vitro [10]. Also, the expression of several components of the transcellular pathway for Ca^2+^ absorption was similar in both genotypes. This includes the jejunal mRNA expression of *Nhe3* and *Atp2b*, the genes coding the apical Na^+^/H^+^ exchanger NHE3 and the basolateral plasma membrane Ca^2+^ ATPase (PMCA2), as well as the jejunal and ileal protein and/or gene expression of the Vdr and calbindin-D28k, despite these genes known to be regulated by calcitriol [24].

Interestingly, while no changes in intestinal paracellular permeability of Ca^2+^ were observed in *Cldn3* KO mice (at least in the jejunum and ileum), they showed diminished urinary excretion of Ca^2+^ upon the high Pi challenge together with high calcitriol, though the mRNA expression of *Trpv5* and *Trpv6* was similar in both genotypes. The cause of elevated calcitriol levels in *Cldn3* KO remains elusive. We speculate that absence of *Cldn3* may indirectly alter intestinal Ca^2+^ absorption and that this effect is even further enhanced under conditions of high dietary Pi intake where Pi can precipitate with Ca^2+^ in the intestine and further reduce its bioavailability.

The inverse correlation between *Cldn3* and calcitriol that we found in our study has already been observed by two other groups. Fujita and colleagues noted enhanced jejunal gene expression of *Cldn3* in VDR-KO mice compared to WT [10], while Kutuzova and DeLuca reported diminished duodenal *Cldn3* expression in rats upon calcitriol treatment [24]. Indeed, the suppressing effects of the VDR-agonist lithocholic acid on claudin-3 were only observed in WT but not in *Vdr*-KO mice [11]. The VDR-mediated calcitriol signaling therefore downregulates *Cldn3*, and our study appears to confirm that the genetic deletion of *Cldn3* reciprocally stimulates calcitriol production in mice. Previously, we demonstrated in mice that calcitriol stimulates only trans- but not paracellular Pi transport [14], consistent with clinical observations in humans. The interpretation that lithocholic acid regulates in a VDR-dependent manner *Cldn3*-dependent paracellular Pi permeability is thus unlikely. Nevertheless, why calcitriol’s effects on Pi and Ca^2+^ metabolism are not present in the *Cldn3* KO mice is currently unknown, as the baseline phenotype of these mice already showed elevated levels of the hormone without any abnormalities in Pi or Ca^2+^ handling. A possible explanation could be a desensitization to calcitriol as a result of the deletion of *Cldn3*, as these mice also showed increased *Vdr* gene expression in the kidney. Further analysis is therefore necessary to elucidate the exact molecular pathway underlying this inverse correlation.

In summary, our study does not provide evidence for altered Pi handling and homeostasis in *Cldn3* KO mice, though calcitriol is increased and associates with hypocalciuria in *Cldn3* KO mice challenged with high dietary Pi.

## Supplementary Information

Below is the link to the electronic supplementary material.

Supplementary file1 (PDF 1164 KB)

## Data Availability

The data that supports the findings of this study are available in the supplementary material of this article.
