# Supplementary material for: Absence of claudin-3 does not alter intestinal absorption of phosphate in mice
Source: Pflugers Arch. 2024 Aug 8;476(10):1597–612. doi: 10.1007/s00424-024-02998-x (PMC11381482; doi:10.1007/s00424-024-02998-x)

Supplementary Table 1. Primers and FAM/TAMRA labelled probes used for quantitative real-time PCR analysis.

**Claudin-12**	FW: GCC TGA CTG GTT CCC TGA AG<br>RV: CAG AGA TAA TCT GGA CCG GCT
**TRPV5**	FW: CGT TGG TTC TTA CGG GTT GAA C<br>RV: GTT TGG AGA ACC ACA GAG CCT CTA<br>Probe: TGT TTC TCA GAT AGC TGC TCT TGT ACT TCC TCT TTG T
**TRPV6**	FW: TTC TGC AGA TGG TTC CAC AG<br>RV: TCT CTG GTG CAC CTC ACA TC<br>Probe: CAG CAG AAG AGG ATC TGG GAA TC
**NHE3**	FW: CAC ACC CCG CCC ATC TAC T<br>RV: CCA GGC ATA CAG CAC TGA CAT T<br>Probe: TGG AGG CGT CTG TCT CAT ATT TCT TGA GGG
**ATP2b4**	FW: GGG CAG TGA GTG AGA ATC AGA<br>RV: AGG TGT GGA TGG AGC TAC GA
**Cyp24a1**	FW: CCA GCG GCT AGA GAT CAA AC<br>RV: CAC GGG CTT CAT GAG TTT CT<br>Probe: TAC GGG CTG ATG ATC CTG GAA GGAC
**Cyp27b1**	FW: CCG ACC CTA CAC TTG TGG AG<br>RV: ATT CTT CAC CAT CCG CCG TT<br>Probe: TCT CAT CAT GGG CAG AGC ACC GTC
**VDR**	FW: GGA TCT GTG GAG TGT GTG GA<br>RV: CAT GCT CCG CCT GAA GAA AC<br>Probe: TAT GAC CTG TGA AGG CTG CA
**HPRT**	FW: TTA TCA GAC TGA AGA GCT ACT GTA ATG ATC<br>RV: TTA CCA GTG TCA ATT ATA TCT TCA ACA ATC<br>Probe: TGA GAG ATC ATC TCC ACC AAT AAC TTT TAT GTC CC
**Claudin-3<br>(for RT-PCR)**	FW: GTA CAA GAC GAG ACG GCC AA<br>RV: GGG CAC CAA CGG GTT ATA GA

Supplementary figure 1

A) Expression of *Cldn3* mRNA (RT-PCR) in intestinal segments of WT mice

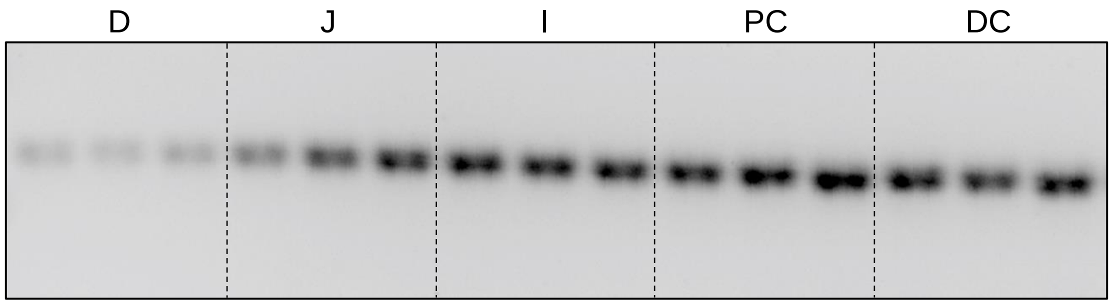

B) Expression of Cldn3 protein in various intestinal segments of WT and *Cldn3* deficient mice

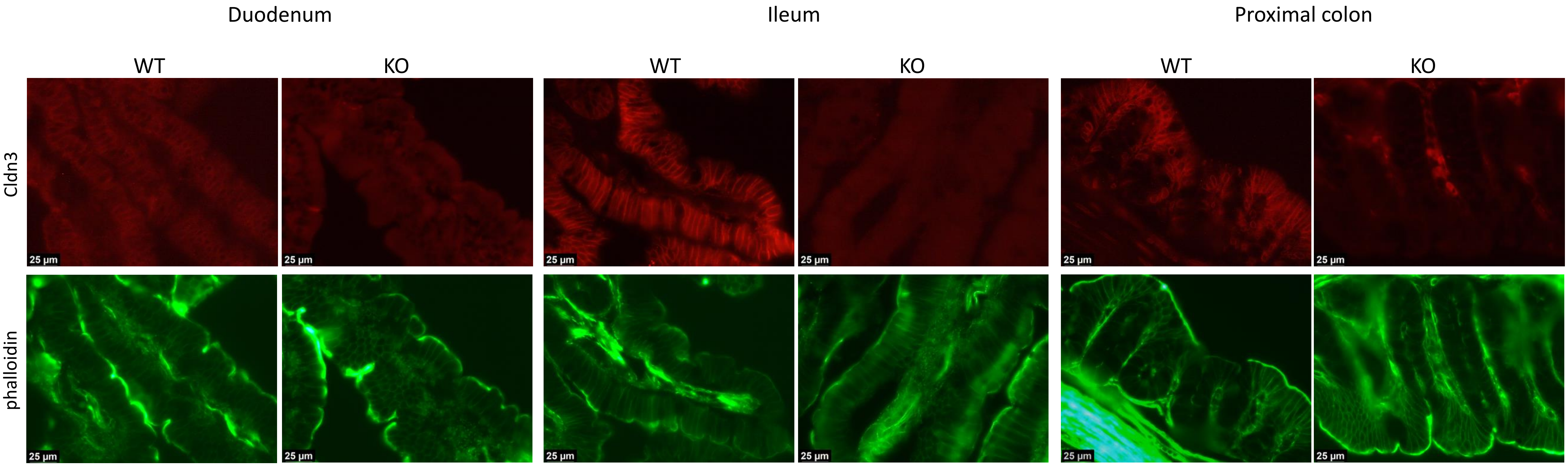

Supplementary figure 2 – mineral parameters under baseline conditions

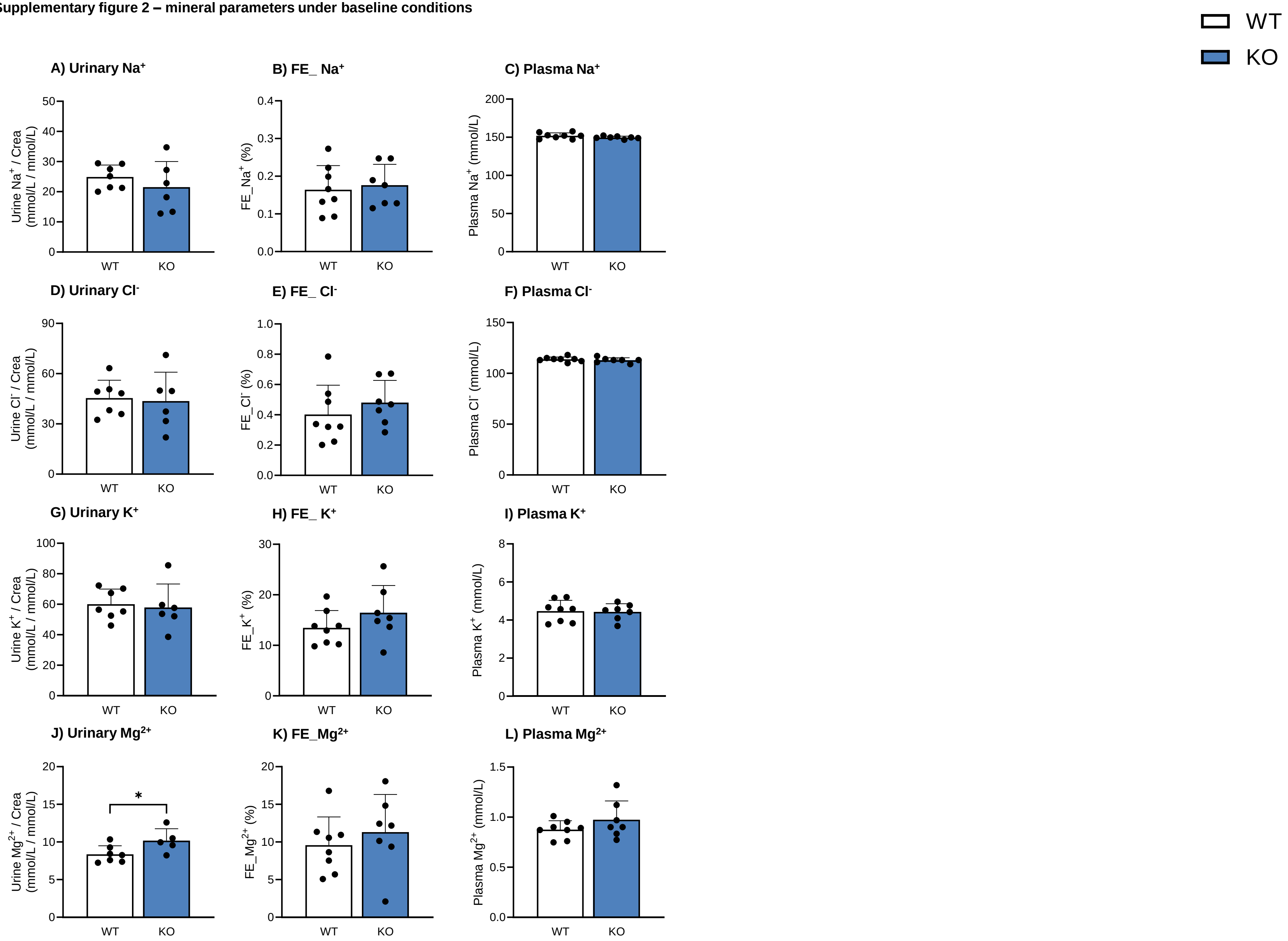

Supplementary figure 3 – mineral parameters under HP conditions

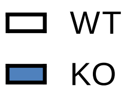

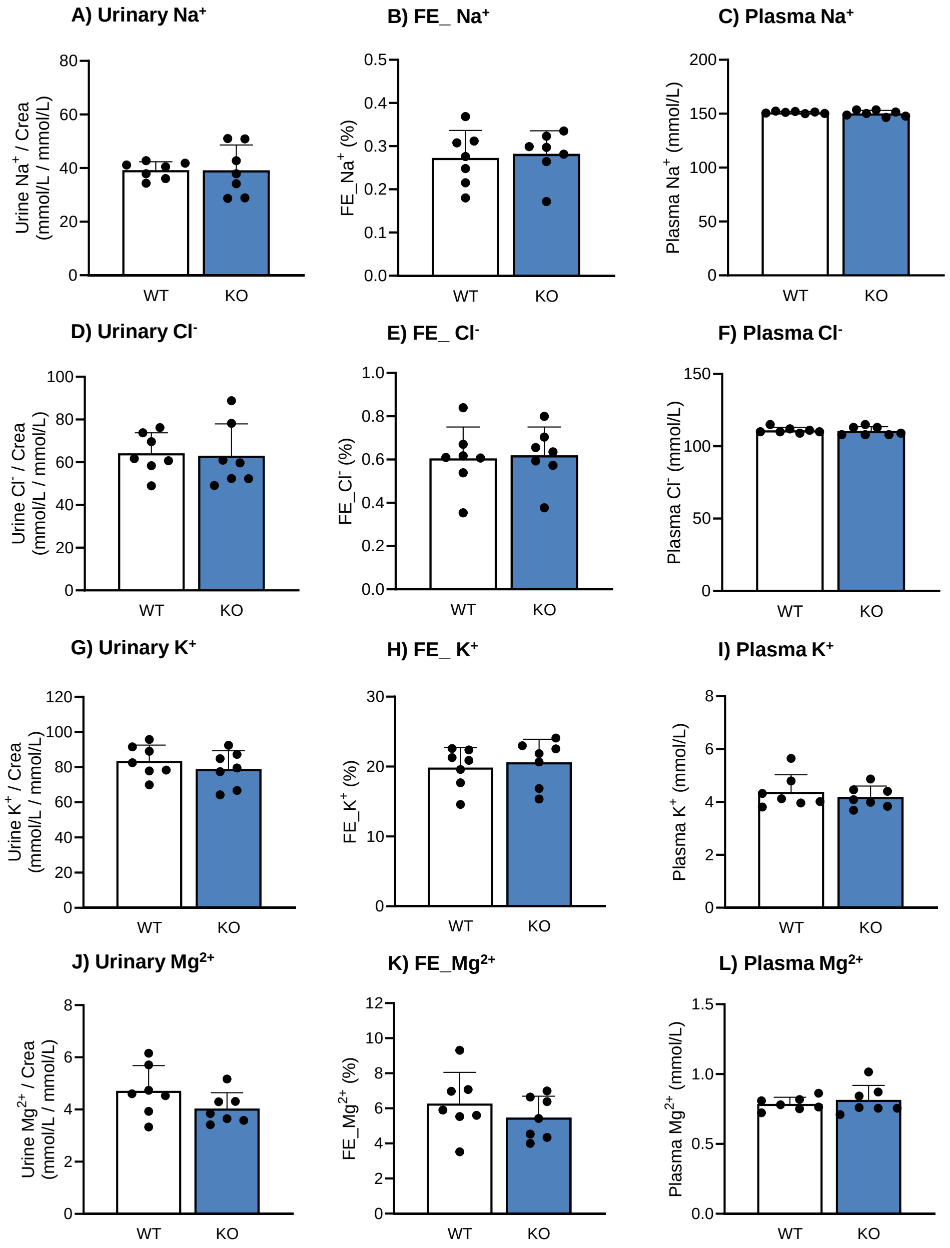

Supplementary figure 4 – Jejunal relative permeabilities upon HP diet

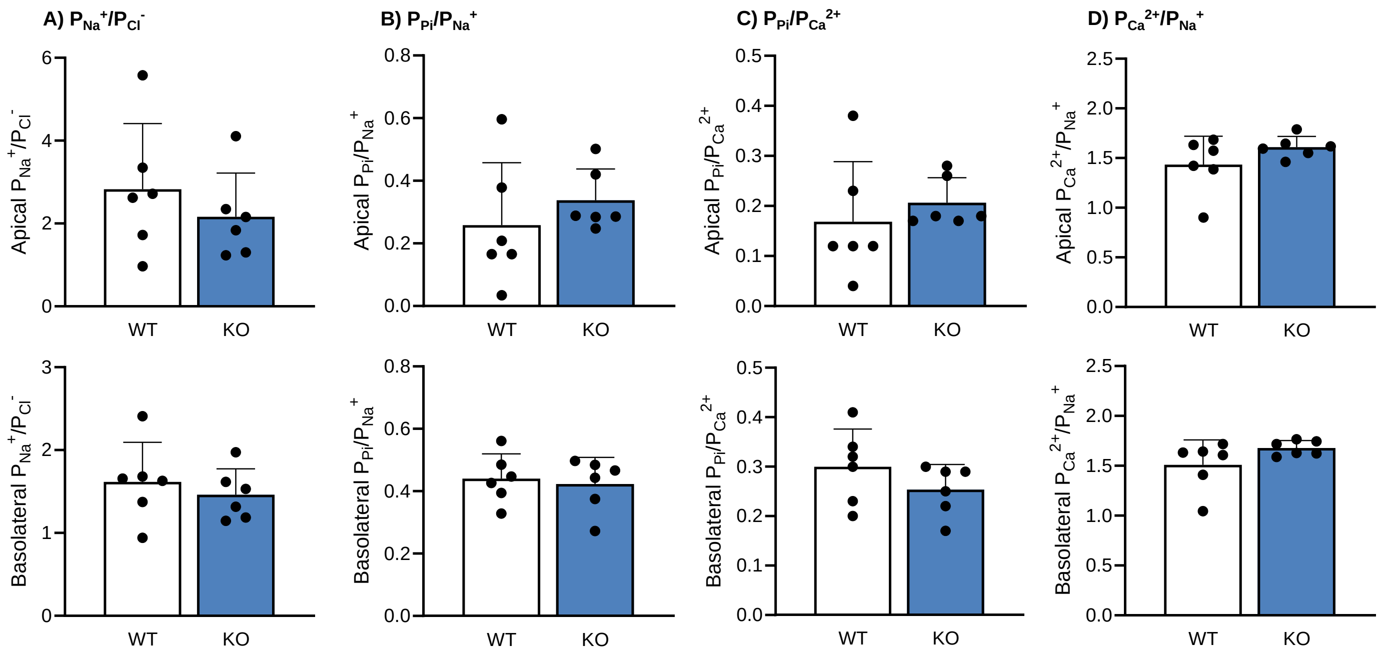

Supplementary figure 5 – Ileal relative permeabilities upon HP diet

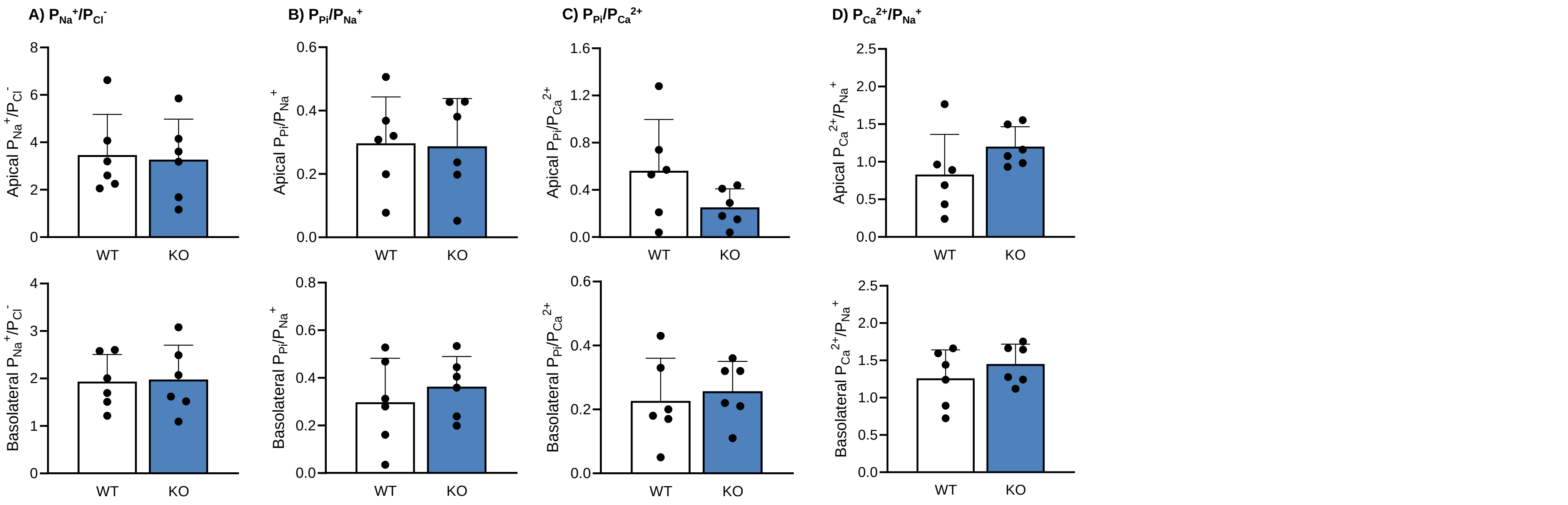

Supplementary figure 6 – HP diet jejunal protein expression

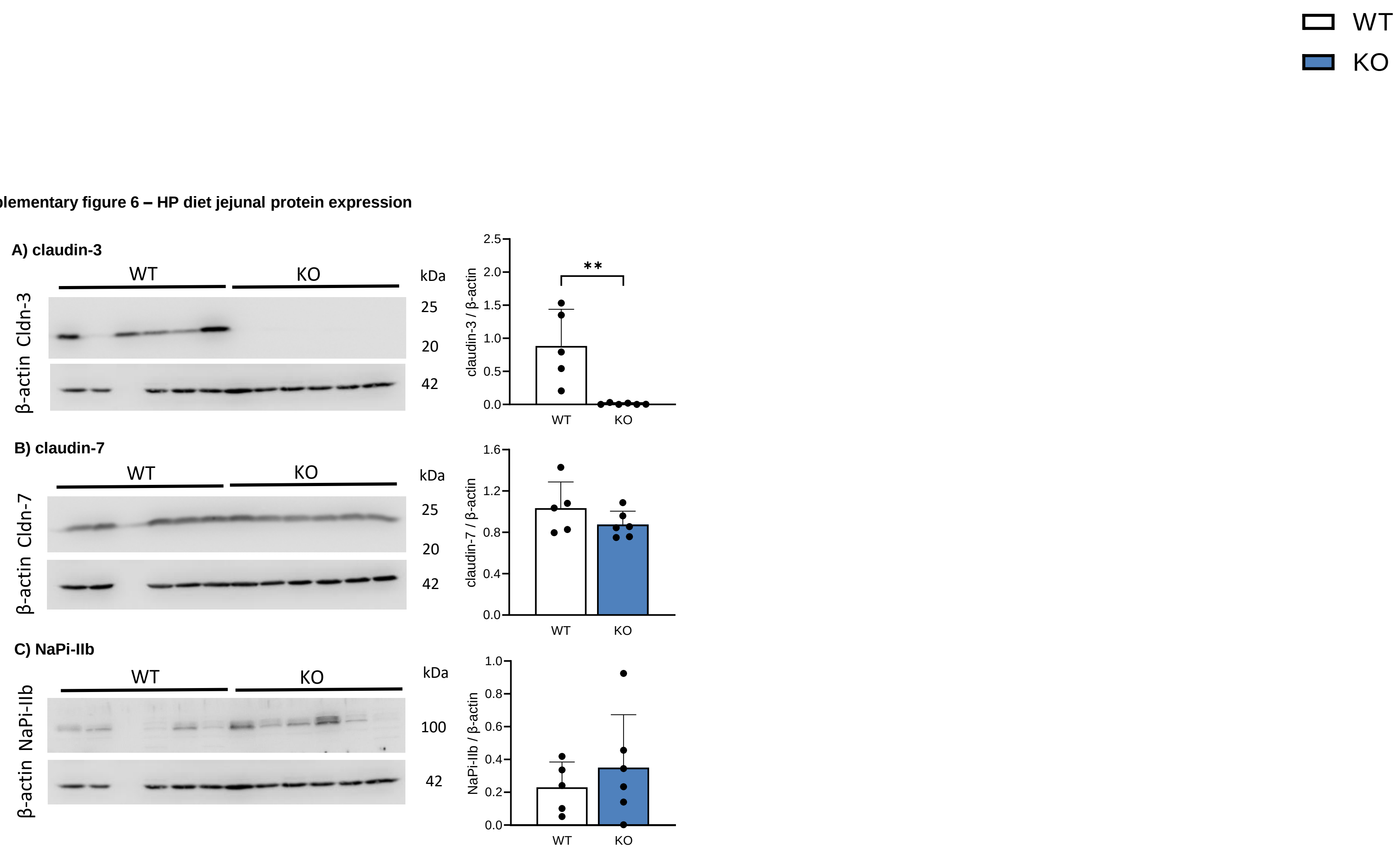

Supplementary figure 7. Intestinal expression of Cldn3 and Cldn7 proteins in WT and *Cldn3* deficient mice

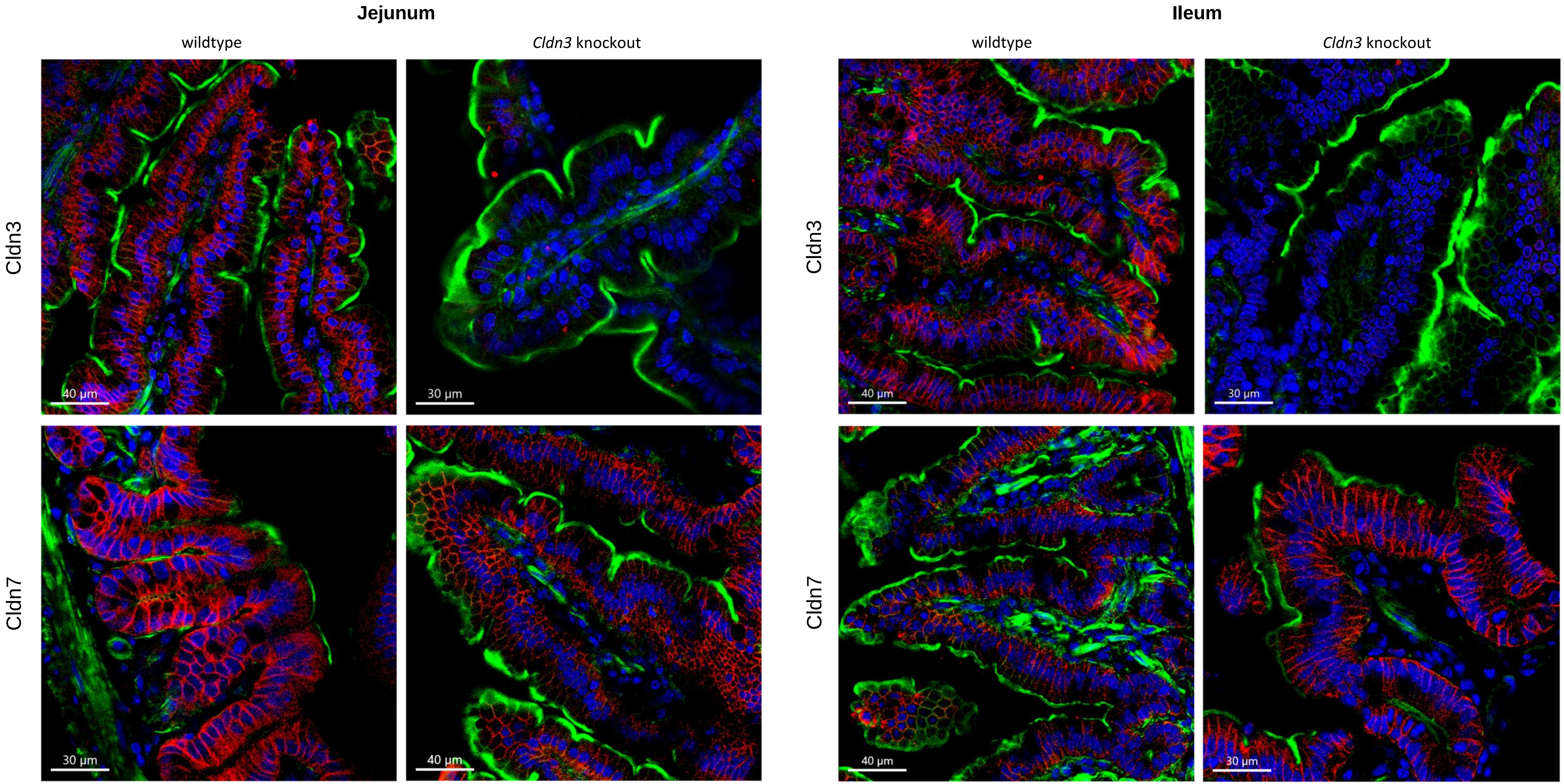

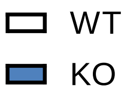

Supplementary figure 8 – renal protein expression

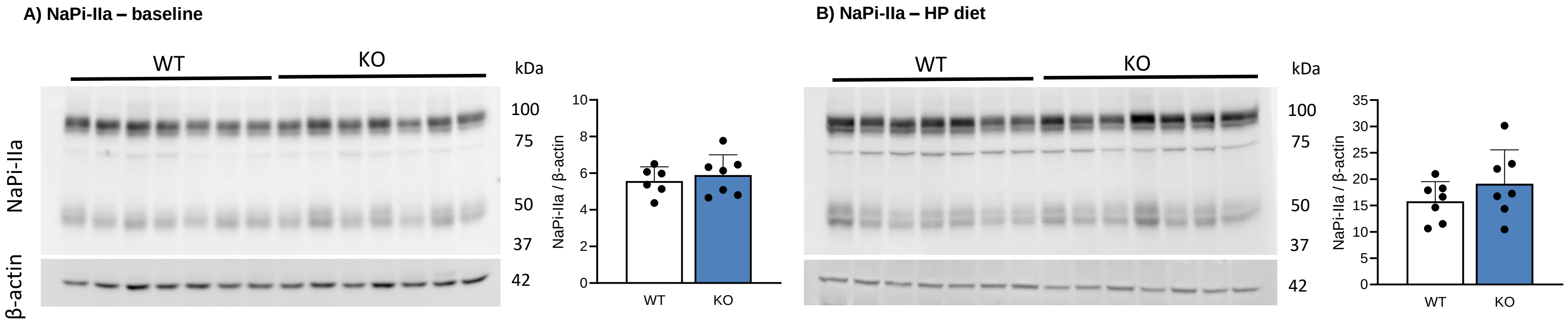

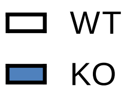

Supplementary figure 9 – HP diet jejunal and ileal protein expression

A) calbindin-D28k - jejunum

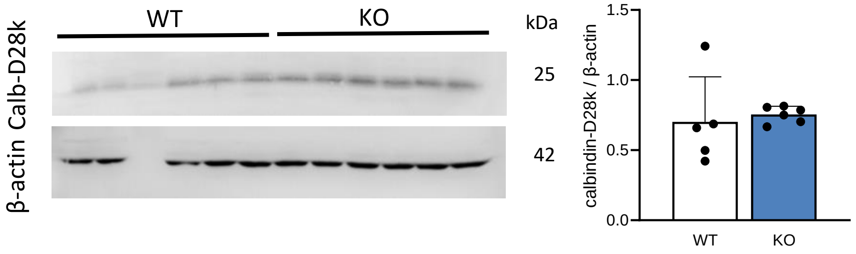

B) calbindin-D28k - ileum

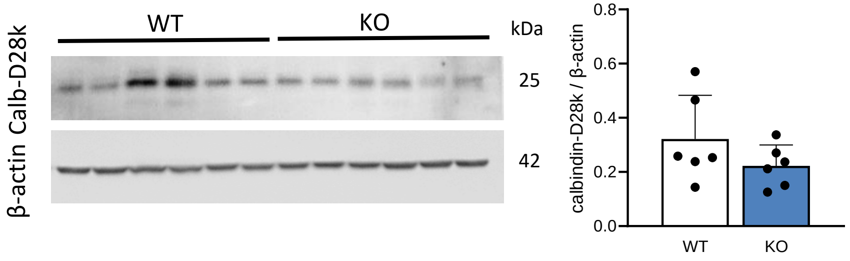

Supplementary figure 10 – jejunal gene expression under baseline and HP conditions

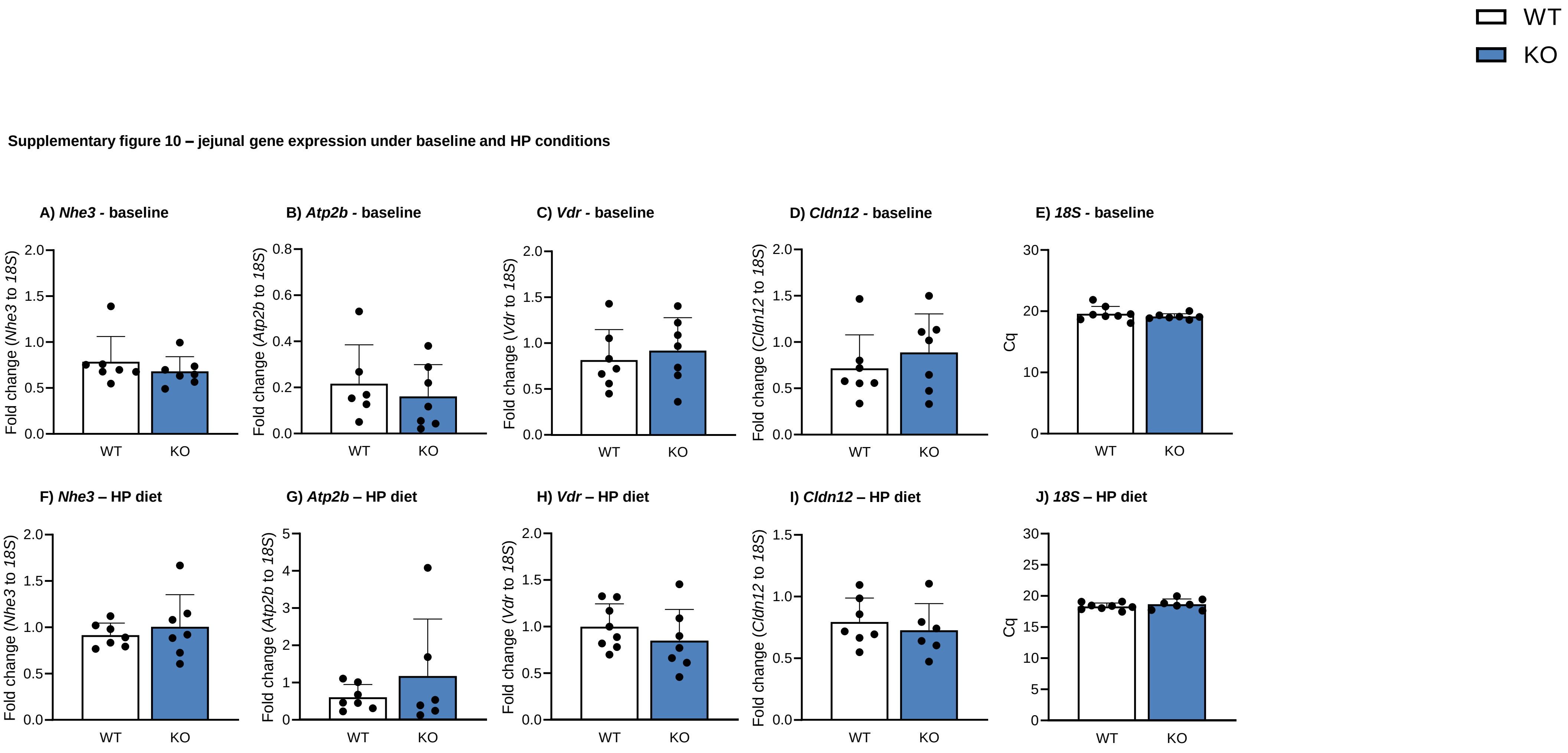

Supplement: Supplementary file 1 — Supplementary file1 (PDF 1164 KB) [file 424_2024_2998_MOESM1_ESM.pdf]
